# Mechanism study of ubiquitination in T cell development and autoimmune disease

**DOI:** 10.3389/fimmu.2024.1359933

**Published:** 2024-03-18

**Authors:** Hui Yu, Wenyong Yang, Min Cao, Qingqiang Lei, Renbin Yuan, He Xu, Yuqian Cui, Xuerui Chen, Xu Su, Hui Zhuo, Liangbin Lin

**Affiliations:** ^1^ Department of Urology, Medical Research Center, Department of Neurosurgery, The Third People’s Hospital of Chengdu, The Affiliated Hospital of Southwest Jiaotong University, The Second Chengdu Hospital Affiliated to Chongqing Medical University, Chengdu, China; ^2^ College of Medicine, Southwest Jiaotong University, Chengdu, China

**Keywords:** ubiquitination, T cell development, self-tolerance, Treg, E3 ubiquitin ligase, TEC, autoimmune disease

## Abstract

T cells play critical role in multiple immune processes including antigen response, tumor immunity, inflammation, self-tolerance maintenance and autoimmune diseases et. Fetal liver or bone marrow-derived thymus-seeding progenitors (TSPs) settle in thymus and undergo T cell-lineage commitment, proliferation, T cell receptor (TCR) rearrangement, and thymic selections driven by microenvironment composed of thymic epithelial cells (TEC), dendritic cells (DC), macrophage and B cells, thus generating T cells with diverse TCR repertoire immunocompetent but not self-reactive. Additionally, some self-reactive thymocytes give rise to Treg with the help of TEC and DC, serving for immune tolerance. The sequential proliferation, cell fate decision, and selection during T cell development and self-tolerance establishment are tightly regulated to ensure the proper immune response without autoimmune reaction. There are remarkable progresses in understanding of the regulatory mechanisms regarding ubiquitination in T cell development and the establishment of self-tolerance in the past few years, which holds great potential for further therapeutic interventions in immune-related diseases.

## Introduction

**Figure 1:**
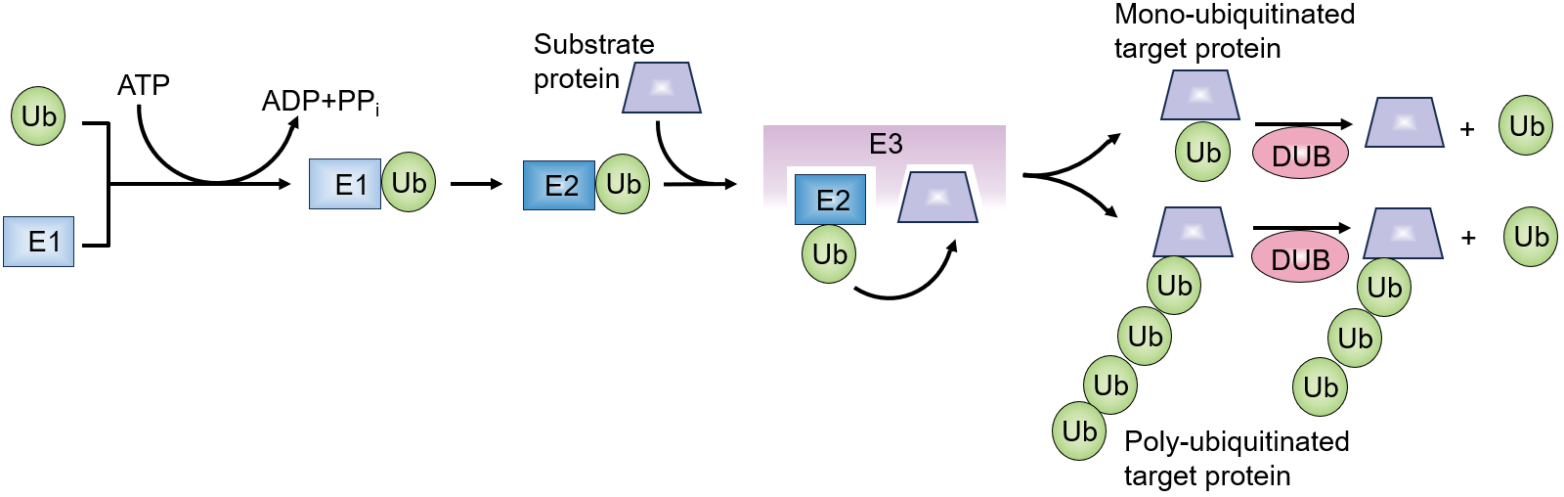
Overview of ubiquitination process. Ubiquitin molecule is activated in an ATP-dependent manner by an ubiquitin-activating enzyme (E1). The activated ubiquitin is then transferred to an ubiquitin-conjugating enzyme (E2). Ubiquitin-protein ligases (E3 ligases) bind the ubiquitin-E2 complex and substrate proteins, facilitating the transfer of ubiquitin from E2 to the target substrate. This process results in the covalent attachment of ubiquitin to the substrate protein. Deubiquitinating enzymes (DUBs) can cleave the isopeptide bond between ubiquitin and substrate proteins, reversing the modification.

Ubiquitination is a post-translational mechanism of protein modification involved in multiple signaling pathways (1, 2). The 76-amino acid ubiquitin is covalently conjugated to lysine (K) residues of target proteins catalyzed by ubiquitin-activating (E1), ubiquitin-conjugating (E2), and ubiquitin-ligating (E3) enzymes (3, 4) (**Figure 1**). Ubiquitin molecules are conjugated to target proteins either singly (monoubiquitination) or in the form of polyubiquitin chains. Polyubiquitin chains are formed by isopeptide bond connection between the carboxyl-terminal glycine residue of ubiquitin and seven internal K residues (K6, K11, K27, K29, K33, K48, K63) or the amino-terminal methionine (M1) of another ubiquitin (5). The various ubiquitin chains play different roles in cellular process while K48- and K63-linked ubiquitin chains are most extensively studied. K48- and K11-linked ubiquitin chains mainly target substrates for degradation by 26s proteasome (6). K63- and M1-linked ubiquitin chains mainly induce signal transduction and DNA repair. Deubiquitinases (DUBs) mediate the reversible modification by cleaving the conjugated ubiquitin from target peotein (7).The substrate specificity of ubiquitination is mainly determined by E3s, which mediate the binding of ubiquitin molecules to target proteins directly or catalyze the transfer of ubiquitin molecules from E2 conjugating enzymes to substrate proteins (5, 8). Over 700 mammalian E3 ubiquitin ligases were recognized (3). The most studied E3 ligase subfamilies are really interesting new gene- (RING-) and homologous to E6AP C terminus- (HECT-) domain containing ligases (9). E3s with RING domain catalyze the transfer of ubiquitin from E2 to substrate (10), while E3s with HECT domain transfer ubiquitin to substrate through covalent thioester intermediate between ubiquitin and the cysteine residue of HECT domain (4, 11).

**Figure 2:**
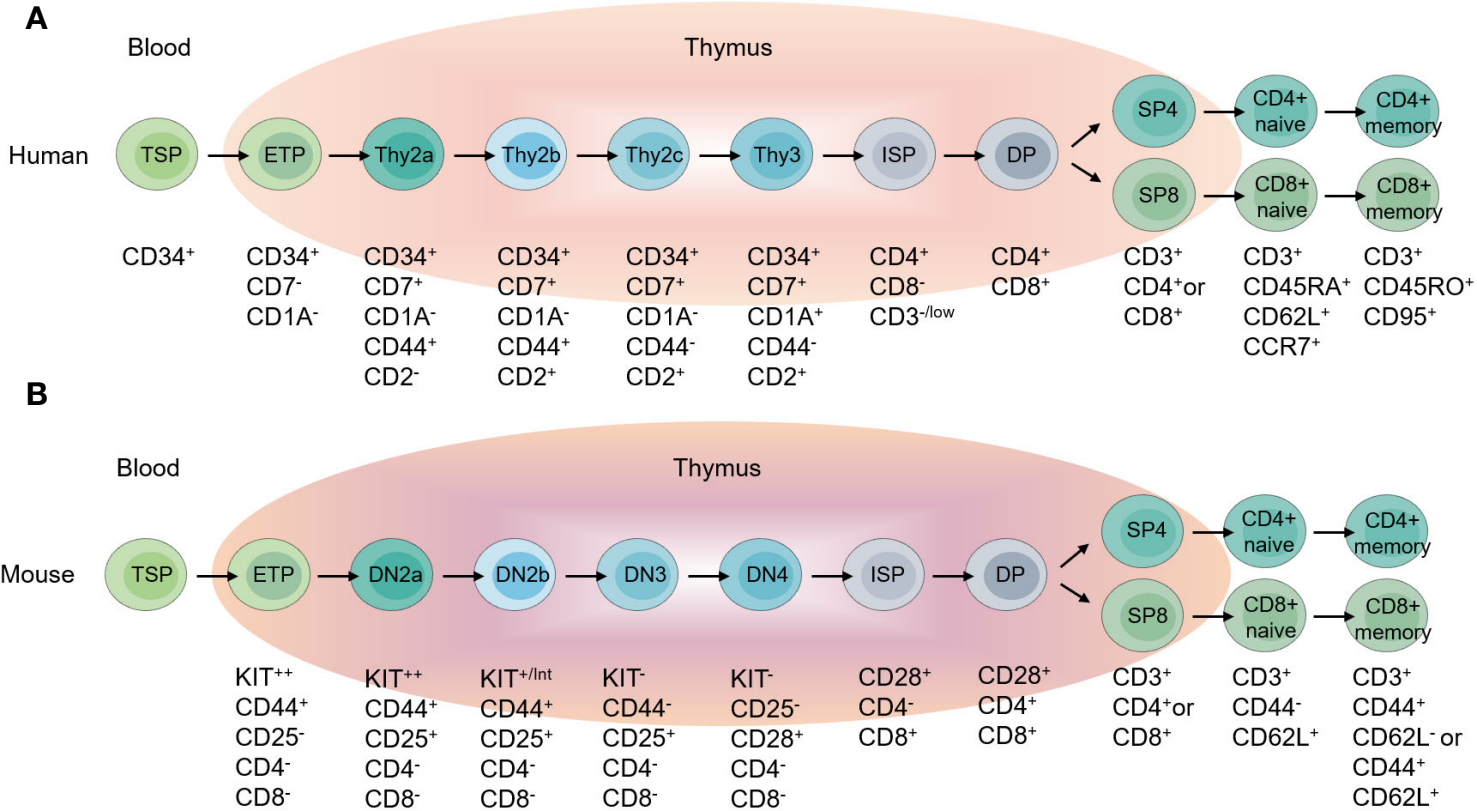
Overview of T cell development. **(A)** Different stages of T cell development in the human thymus, and peripheral T cell subpopulations. **(B)** Different stages of T cell development in the mouse thymus, and peripheral T cell subpopulations.

T cells are implicated in multiple immune processes, including immune response to pathogens, tumors and also in inflammation, immune homeostasis, immune memory and self-tolerance maintenance (12, 13). Fetal liver or bone marrow derived thymus-seeding progenitors (TSPs) settle in thymus, developing into early thymic progenitors (ETPs), which give rise to γδT and αβT cells (13–15). Under the driving of the specialized thymic microenvironments, ETPs proliferate extensively and undergo several selection processes to mature into distinct functional T cell lineages. For the best-studied conventional CD4^+^ and CD8^+^ αβT cells, ETPs develop into non-self-reactive T cells with the ability to recognize MHC-antigen complexes along the CD4^-^CD8^-^ double negative (DN), CD4^+^CD8^+^ double positive (DP) and CD4^+^ or CD8^+^ single positive (SP) stages (16–18) (**Figure 2**). During thymocyte development, the strength of TCR-peptides-MHC interaction decides the cell fate (19, 20). Weak or absent TCR-peptides-MHC interaction leads to cell death, and low or moderate affinity of TCR-peptides-MHC interaction promotes T cell development through positive selection. High-affinity TCR-peptides-MHC interaction triggers the death of self-reactive thymocytes during negative selection. The combined positive selection and negative selection ensure the production of the conventional naïve CD4^+^ and CD8^+^ αβT cells. However, some self-reactive thymocytes survive and develop into regulatory T cells (Tregs) or unconventional T cells including invariant natural killer T (iNKT) cells, natural TCRαβ^+^ CD8αα^+^ intraepithelial T cells and natural T helper 17 (nTh17) cells, through a process called agonist selection (21, 22).

**Figure 3:**
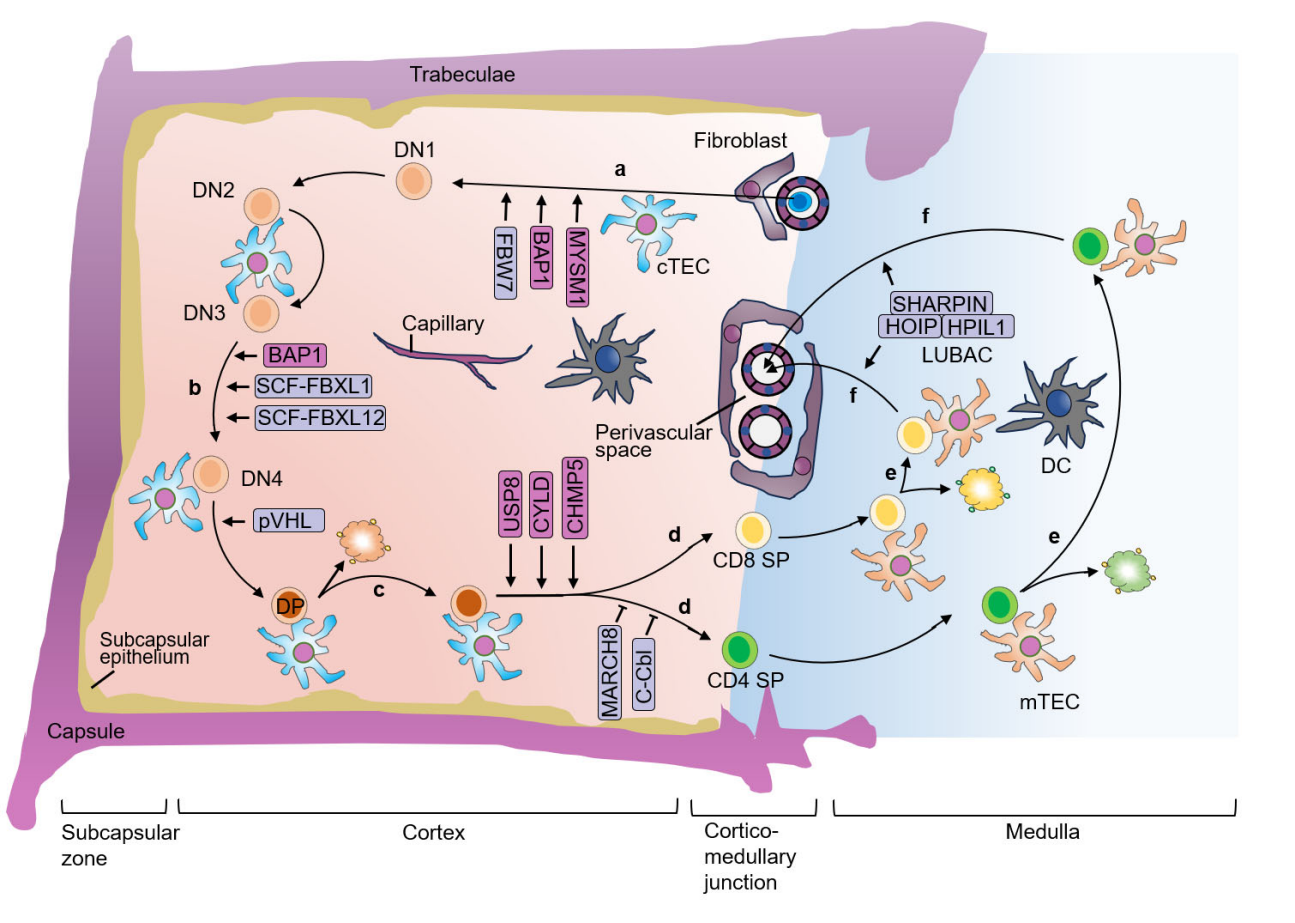
E3 ubiquitin ligases and DUBs in different stages of thymocyte development. **(A)** In the postnatal thymus, T-lymphoid progenitor cells that circulate in the blood migrate into the tissue of the thymus through blood vessels that are concentrated around the cortico-medullary junction. **(B)** Thymocytes proliferate rapidly after TCR β-chain rearrangement when the pre-TCR is expressed, allowing the thymocytes to undergo β-selection. **(C)** cTEC express MHCI or MHCII molecules, and thymocytes that successfully rearrange the TCRα chain recognize the autoantigen bound to MHCI or MHCII with low or medium affinity, thus activating TCR, and the thymocytes undergo positive selection. **(D)** After positive selection, DP cells develop into CD4^+^ cells that recognize MHCII molecules or CD8^+^ SP cells that recognize MHCI molecules. **(E)** The SP thymocytes undergo negative selection, and only T cells that do not recognize self-antigens survive, establishing self-tolerance and further differentiate into mature CD4^+^ or CD8^+^ SP cells. Autoreactive TCRs recognize MHC-autoantigen peptides presented by mTEC and DC with high affinity, leading to death and clearance. **(F)** The surviving SP cells undergo further maturation until they are ready to exit the thymus. DN, double-negative; DP, double-positive; SP, single-positive; cTEC, cortical thymic epithelial cell; mTEC, medullary thymic epithelial cell; DC, dendritic cell; TCR, T cell receptor; MHC, major histocompatibility complex.

**Figure 4:**
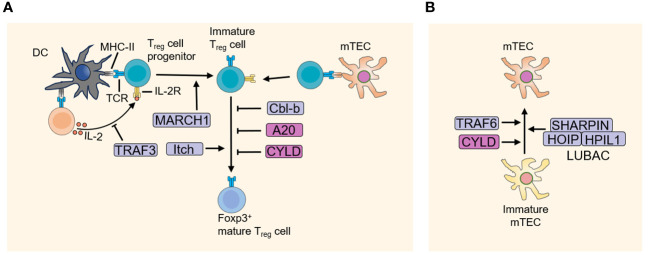
E3 ubiquitin ligases and DUBs in the development of Treg cells and mTEC. **(A)** The thymus niche for Treg cell development is defined by DC and mTEC that presents a specific, possibly tissue-restricted antigen-derived agonist self-peptide. Treg cell progenitors receive strong TCR and IL-2 signaling, promoting its development into mature FOXP3^+^ Treg cells. And E3 ubiquitin ligases and DUBs play a crucial role in the development of Treg. **(B)** E3 ubiquitin ligases and DUBs regulate the development of mTEC. DC, dendritic cell; DUBs, Deubiquitinating enzymes; mTEC, medullary thymic epithelial cell.

The ordered and sequential differentiation and selection during thymocyte development are tightly regulated and correlated with the generation of non-self-reactive T cells with functional TCRs. In the past few decades, increasing studies demonstrated the fundamental role of ubiquitination regulation in T cell development. Figuring out the ubiquitination regulatory mechanisms of T cell development not only enhances our understanding of how T cell fate is determined, but also provides potential new therapeutic approaches for T cell dysfunction-associated and autoimmune diseases. It is worth noting that drugs targeting the ubiquitin-proteasome system has shown promise in the treatment of autoimmune disorders and cancers, such as bendamustine (targeting LUBAC), thalidomide (targeting CRBN), and mitoxantrone (targeting USP11, USP15) et al. (23–25). In this review, we discuss the importance of ubiquitination in T cell development (**Figure 3**) and self-tolerance establishment (**Figure 4**), with an emphasis on the mechanism studies of T cell progenitors, thymic selections, proliferation, apoptosis, Treg development and TEC functions.

## Ubiquitination regulation of T cell progenitors

CD34 serves as a marker for human stem cells and progenitor cells, labeling T cell precursors in the thymus (26). Human DN thymocytes are divided into three subsets based on the expression of CD34, CD7, and CD1a, which correspond to the developmental order: CD34^+^CD7^-^CD1a^-^ (Thy1), CD34^+^CD7^+^CD1a^-^ (Thy2), and CD34^+^CD7^+^CD1a^+^ (Thy3) (27–31)(**Figure 2**). The expression of CD1a marks T cell lineage commitment, and Thy1 cells are the fewest and the earliest T cell precursors with multi-lineage differentiation potential including T cells, B cells, NK cells and myeloid cells *in vitro (* 27, 30, 32). Recent transcriptomics studies revealed the heterogeneity of Thy2 cells. Based on the expression of CD44 and CD2, Thy2 cells are divided into three subsets, CD44^+^CD2^-^ (Thy2a), CD44^+^CD2^+^ (Thy2b) and CD44^-^CD2^+^ (Thy2c) (33). CD2 expression marks the stage of T lineage commitment and the multi-lineage differentiation potentials. CD2^-^ Thy2a cells are most similar to Thy1 cells, with multi-lineage differentiation potential including T, B, NK and myeloid cells *in vitro*. CD2^+^ Thy2b cells exhibit T, NK and myeloid cell differentiation potential, but no longer B cell differentiation capability (33). Downregulation of CD44 represents lineage commitment of T cells, thus Thy2c and Thy3 are T-lineage committed and have no other lineages differentiation potentials (33, 34). CD34^+^CD38^-^ cells have also been shown to contain the earliest progenitors in thymus. CD38 is expressed before CD7, and in CD7^-^ cells, more than half of the cells express CD38 (30, 35).

In mice, DN cells are divided into four populations: DN1, DN2, DN3, and DN4, with surface marker expression profiles of CD44^+^CD25^-^, CD44^+^CD25^+^, CD44^-^CD25^+^, and CD44^-^CD25^-^ respectively (36–39)(**Figure 2**). DN1 contains mouse ETP (CD44^+^KIT^hi^CD25^-^), possessing multi-lineage differentiation potential (39, 40). DN2 cells contain DN2a and DN2b populations. DN2a also has multi-lineage differentiation potential, capable of differentiating into T, NK and ILC lineages. DN2b cells have been committed to T cell lineage and lose differentiation potential into other lineages (41–43). The onset of the rearrangement of gene encoding T cell receptor β (*Tcrb*), γ (*Tcrg*), and δ (*Tcrd*) occurs during the DN2a to DN2b transition (44, 45). From DN2 to DN3, γδT and αβT cell fate are determined (46, 47).

## BAP1

BRCA1-associated protein-1 (BAP1) is a member of the ubiquitin C-terminal hydrolase (UCH) subfamily of DUBs, the function of which in cancers is well studied (48, 49). BAP1 deletion in mice leads to severe thymic atrophy in both the cortex and medulla area. ETPs, DN, ISP and DP thymocytes are decreased in tamoxifen-induced *Bap1* deletion mice (*Bap1* ^fl/fl^ *Rosa26* ^CreERT2^) (50). Further study reveals that BAP1 deficiency results in the expansion of hematopoietic progenitor cells in bone marrow, but inhibits the development of B cells. *In vitro* culture experiments show that BAP1-deficient thymic precursors progress normally at DN1, DN2 and DN3 stages, but blocked at DN3 stage. In the absence of BAP1, DN cells lose the capability of transition to DP stage (50). Reconstruction of *Bap1* ^fl/fl^ *Rosa26* ^CreERT2^ lineage-negative bone marrow progenitors with WT or mutant BAP1 (C91A, with no catalytic activity) shows that the mutant BAP1 (C91A) is not able to restore T cell development, suggesting the deubiquitinating activity of BAP1 is necessary for T cell differentiatio (50). Mechanism study shows decreased phosphorylation of H3S10 and enhanced ubiquitination of H2AK119 in BAP1-deficient cells, which is associated with G_2_-M transition in cell cycle, suggesting BAP1 regulates cell proliferation in thymocytes by facilitating the deubiquitination of H2AK119 and allowing G_2_-M transition (50).

## FBW7

The RING-containing E3 ubiquitin ligase F-box and WD repeat domain-containing 7 (FBW7, also called FBXW7) functions as the substrate recognition component of the Skp-cullin-F-box (SCF) E3 ubiquitin ligase. Knockout of FBW7 is embryonically lethal (51, 52). FBW7 mediates the ubiquitination and degradation of NOTCH1 and c-MYC, thereby involving in hematopoietic cell development (53–55). Hematopoietic-specific knockout of FBW7 impairs the self-renewal of hematopoietic stem cells (HSCs). *Fbw7^-/-^* HSCs have defective cell cycle quiescence, failing to enter the resting state and thereby impacting self-renewal (56). As total c-MYC deficiency completely abrogates HSC differentiation, the single Myc allele was deleted in *Fbw7^-/-^* Lin^-^Sca-1^+^c-Kit^+^ (LSK) cells, and resulted in restored LSK numbers, cell-cycle status and *in vitro* self-renewal capacity, indicating the HSC reduction in *Fbw7^-/-^* mice is due to accumulation of c-MYC (57). Notably, hematopoietic-specific knockout of FBW7 also impairs T cell development, with significantly reduced numbers of thymocyte at 4 weeks after polyI-polyC-induced deletion of *Fbw7*. In hematopoietic-specific FBW7 knockout mice, ETPs and DN cells are reduced significantly, while DN2 and DN3 thymocytes are nearly completely blocked (56).

## MYSM1

Myb-like SWIRM and MPN domain containing 1 (MYSM1) belongs to the metalloprotease protein family and have deubiquitinase activity (58, 59). By coordinating chromatin structure and transcriptional programs, MYSM1 plays integral roles in hematopoietic stem cell activity, blood cell development, and immune system (58, 60, 61). In addition, cytosolic MYSM1 is shown to regulate innate immune signaling pathways by promoting the deubiquitination of tumor necrosis factor receptor-associated factor 3 (TRAF3), 6 (TRAF6), and receptor interacting protein 2 (RIP2) (62, 63). MYSM1 also functions in the early development stage of thymocyte (64). *Mysm1* ^−/−^ mice show severely hypoplastic thymus due to decreased ETPs in context with lymphoid-primed multipotent progenitor (LMPP) defects in bone marrow, partial block of DN1-DN2 transition, and increased apoptosis of thymocytes (64). Further mechanism study reveals that increased apoptosis is correlated with the upregulation of p19^ARF^ in *Mysm1*-deficient thymocytes, which is an important regulator of the p53 tumor suppressor protein. *Mysm1*-deficient thymocytes exhibit increased expression of p53 and p53 target genes such as *Bax* and *p21^Waf/Cip^*, as well as decreased expression of pro-survival proteins MCL-1 and BCL-X_L_. In *p53* ^-/-^ *mysm1* ^-/-^ double-deficient mice, defects caused by *Mysm1* deficiency are almost completely restored, including reduction of lymphoid-primed multipotent progenitors and decreased number of thymocytes, confirming the involvement of p53-mediated apoptotic pathway in early thymocytes development (64).

## Ubiquitination regulation of thymic selection

During development, T cells undergo TCR rearrangement and selection, eventually becoming mature, self-tolerant CD4^+^ or CD8^+^ T cells (13). TCR is divided into αβTCR and γδTCR. The variable (V), diversity (D), and joining (J) gene segments of the α, β, γ, and δ chains undergo rearrangement, forming a diverse TCR repertoire in a process called V(D)J recombination (13, 14, 65). V(D)J recombination is mediated by the recombination activating gene 1 (RAG1) and 2 (RAG2) (66). In human thymus, TCR rearrangement follows the sequential order of *TCRD* > *TCRG* > *TCRB* > *TCRA (* 35, 67, 68). *TCRD* gene rearrangement is initiated at the CD34^+^CD38^-^CD1a^-^ stage (35). *TCRB* gene rearrangement begins at the CD34^+^CD38^+^CD1a^-^ stage, followed by the expression of CD4 and the immature single positive CD4^+^ T cells (CD4 ISP) (35). CD4 ISP have the potential to develop into αβT and γδT cells (13, 37). β-selection occurs at CD34^+^CD38^+^CD1a^+^ stage in human and DN3 stage in mice (40, 47), which is an important checkpoint in αβT cell development. At this point, the rearranged β chain pairs with pre-TCRα and CD3 to form the pre-TCR complex (47, 69), which provides survival, proliferation and allelic exclusion signals without ligand binding (70–74). Thymocytes with a successfully rearranged TCRβ chain are selected for further differentiation, while thymocytes with failed TCRβ chain rearrangement undergo apoptosis (75, 76). Pre-TCR signaling induces a burst of proliferation, the rearrangement of TCRα chain and silence of TCRδ chain, as well as differentiation of CD4 ISP to DP cells (77–79). After entering the DP stage, the expression of pre-TCR is rapidly downregulated. DP thymocytes express CD4 and CD8. CD8 is a heterodimer consisting of α and β chains. CD8α chain expression precedes β chain, and thymocytes expressing CD8α chain are termed as early DP cells, which further develop into CD4^+^CD8α^+^β^+^ DP cells (47, 80–82).

Pre-TCR induces rearrangement of TCR α chain. The successfully rearranged α chain combines with the β chain to form the αβTCR (35, 83). Thymocytes with successfully rearranged TCR α chain recognize self-antigens presented by MHCI or MHCII with low or moderate affinity, thus activating the TCR signaling to provide proliferation and survival signals, which is known as positive selection (84, 85). Positively selected thymocytes upregulate expression of the early activation molecules CD69, CC-chemokine receptor 7 (CCR7), and subsequently CD27, while the expression of *RAG1* and *RAG2* is downregulated (86–90). Through binding of CCR7 expressed by thymocytes and CC-chemokine ligand 21 (CCL21) expressed by medullary TECs (mTECs), positive-selected thymocytes migrate to the thymic medulla for negative selection (87–89). High-affinity TCR-self-peptides-MHC interaction triggers cell death by apoptosis to establish self-tolerance, which is known as negative selection. After positive and negative selection, T cells acquire the ability to recognize alloantigens-MHC without being self-reactive, differentiating into mature CD4^+^ or CD8^+^ SP cells with naïve marker CD45RA^+^CCR7 +  (84, 91), and then enter the periphery to perform immune functions (**Figure 2**).

## Cbl

Casitas B-lineage lymphoma (Cbl) belongs to RING-containing E3s. Cbl-b and c-Cbl are highly homologous expressed in hematopoietic cells. Both c-Cbl and Cbl-b are expressed in thymocytes and mature peripheral T cells, with c-Cbl expressing higher in DP cells than mature T cells, while Cbl-b is expressed higher in mature CD4 and CD8 T cells, suggesting c-Cbl and Cbl-b play different roles in T cell development and functions (92). Cbl-b deficient mice have normal thymic T cell development, while c-Cbl deletion leads to significant increase of CD4^+^ SP thymocytes (93). Using H-Y TCR transgenic mice, it was found that loss of c-Cbl promotes positive selection of CD4^+^ T cells without affecting CD8^+^ T cells, meanwhile, peripheral c*-Cbl^-/-^* CD4^+^ T cells are less responsive to antigenic stimulation (93). These results indicate that c-Cbl selectively inhibits positive selection of CD4^+^ but not CD8^+^ T cells. Mechanistic studies reveal that c-Cbl deletion enhances TCR activation in thymocytes, with increased phosphorylation of ZAP70 and downstream ERK1/2 activation (93). *c-Cbl^-/-^* DP cells expressed higher level of CD3, CD5, and CD69 molecules in proportions (93), indicating increased DP cells undergoing positive selection after c-Cbl deletion. Therefore, it is hypothesized that c-Cbl regulates TCR signaling by controlling ZAP70 and ERK activation, thereby promoting positive selection of thymocytes. However, whether c-Cbl directly regulates the ubiquitination of ZAP70 has not been demonstrated. Considering the enhanced CD3 and CD4 expression after c-Cbl deletion, it is possible that c-Cbl deletion promotes TCR-MHC complex interactions and thus positive selection of CD4^+^ T cells (93). In addition, another homologue Cbl promotes ubiquitination of TCRζ chain with its functional variant Src homology 2 domain and intact RING finger, while ZAP70 acts as an adaptor protein. Disrupt the interaction of TCRζ/ZAP70 or ZAP70/Cbl reduces the ubiquitination of TCRζ, by which Cbl negatively regulates the activation of TCR and positive selection during T cell development (94).

CD4 and CD8 lineage commitment is highly dependent on MHCII and MHCI, respectively. Thymocytes with TCR recognizing MHCII develop into CD4^+^ T cells and with TCR recognizing MHCI choose CD8^+^ T cell fate (95). Simultaneous deletion of c-Cbl and Cbl-b by *Lck-Cre* enhances thymic negative selection (96). c-Cbl and Cbl-b double deficient thymocytes still generate CD4^+^ and CD8^+^ cells in the absence of MHCI and MHCII molecules (96). Further studies find that c-Cbl and Cbl-b double deficient DP thymocytes have high expression of pre-TCR, as well as spontaneous and constitutive activation pre-TCR signaling (96). Pre-TCR is briefly expressed after TCRβ chain rearrangement in DN cells and downregulated after thymocytes entering DP stage during T lymphocyte development (77). pre-TCRα, TCRβ and CD3 form pre-TCR complex, which is constitutive activated without ligand stimulation to provide proliferation and allelic exclusion signals to mediate the transition of DN to DP stage (97, 98). It has been reported that c-Cbl mediates ubiquitination and degradation of pre-TCR (99), therefore downregulating pre-TCR signaling and enabling normal T cell development in MHC-dependent manner. In the absence of c-Cbl and Cbl-b, pre-TCR signaling fails to downregulate properly, providing constitutive survival and proliferation signals for thymocytes, thus these thymocytes no longer require αβTCR activation signals induced by MHC interaction to generate CD4^+^ or CD8^+^ SP T cells (96, 100). Therefore, by utilizing the constitutive activation signals provided by pre-TCR, thymocytes still develop into CD4^+^ and CD8^+^ T cells in c-Cbl and Cbl-b double-deficient mice.

## CYLD

CYLD is a deubiquitinase that removes K63-linked ubiquitin of TRAF2, TRAF6 and nuclear factor (NF)-kappaB essential modulator (NEMO), thereby blocking NF-κB signaling pathway. CYLD deficient mice (exon 2 deletion, *Cyld^Δ2^*) have impaired thymocyte development, indicated by increased DP and decreased SP cells, leading to significantly reduced peripheral T cells (61). Mechanistic studies reveal that CYLD positively regulates TCR signaling in thymic DP cells through binding and removing K63- and K48-linked ubiquitin chains from active LCK, facilitating LCK recruitment of ZAP70 and promoting thymocyte development (61). However, another study using mice with *Cyld* gene exon 2 and 3 knocked out (*Cyld^Δ2/3^*) showed no effect on thymocyte development, with comparable percentage of DP and SP thymocytes between wild-type mice and *Cyld^Δ2/3^* mice, and these *Cyld^Δ2/3^* mice are more susceptible to colonic inflammation and tumor incidence (101). Molecular mechanism studies reveal that CYLD deletion (exon 2 and 3) enhances NF-κB signaling. *Cyld^Δ2/3^* T cells have increased NEMO ubiquitination and NF-κB activation upon anti-CD3 stimulation (101). Meanwhile, TNF-α activated macrophages also have increased TRAF2 ubiquitination and NF-κB activation in *Cyld^Δ2/3^* mice, indicating CYLD controls NF-κB pathway activation by deubiquitinating NEMO and TRAF2, thereby regulating inflammatory responses (101).

To verify the role of CYLD in T cell development, another study using *Lck-cre* mice to delete exon 9 of *Cyld* gene (*Cyld^Δ9^*) in T cells, which encodes a region essential for deubiquitinase activity of CYLD. Deleting exon 9 of *Cyld* gene inhibits the catalytic activity of CYLD. *Cyld^Δ9^* mice show significantly reduced SP thymocytes and peripheral T cells (102). By verifying the expression of CD69, TCR and CD5, researchers found that positive selection is markedly blocked in *Cyld^Δ9^* mice, and occurrence of apoptosis in DP thymocytes is increased (102). Meanwhile, basal NF-κB and JNK activity is elevated in *Cyld^Δ9^* DP thymocyte. Inactivation of NEMO could restore the NF-κB activity and thymocytes development in *Cyld^Δ9^* mice, indicating CYLD controls NF-κB activation by modulating NEMO, thereby establishing proper activation threshold for thymocytes positive selection (102). The distinct targeting strategies employed in generating the three CYLD mutants result in phenotypic differences in thymocyte development. These differences may arise from the expression of different truncated CYLD proteins following exon deletion and the use of T-cell specific versus germ-line deletion strategies. The germ-line deletion approach may introduce interference from non-T cells, further contributing to phenotypic variations.

## CHMP5

CHMP5 is an endosomal sorting complex required for transport (ESCRT) proteins, which are mainly involved in multivesicular body formation (103, 104).Indeed, CHMP5 promotes thymocyte survival during positive selection through post-translational modifications. DP cells express the highest mRNA levels of *Chmp5* among the different thymocyte subsets. *Cd4-cre* mediated *Chmp5* knockout in T cells leads to significant thymic disruption, with markedly reduced CD4^+^ and CD8^+^ SP thymocytes and mature CD24^lo^TCRβ^hi^ T cells, along with greatly diminished αβT cells in peripheral lymphoid organs. The remaining T cells display a CD44^hi^ phenotype, indicating severely impaired T cell development (105). Positively selected thymocytes initiate anti-apoptotic programs including expression of the pro-survival protein Bcl-2 (106–108). Bcl-2 is inactivated and degraded upon oxidation (109). It was revealed that positive selection induces CHMP5-USP8 interaction, thereby maintaining CHMP5 protein stability. CHMP5 in turn inhibits oxidation and degradation of Bcl-2, ensuring survival of positively selected thymocytes (105).Deletion of the pro-apoptotic gene *Bim* or transgenic overexpression of Bcl-2 can reverse the T cell development defects caused by CHMP5 deficiency (105). Further studies show CHMP5 binds USP8 in CD69^+^TCRβ^hi^ DP cells but not in CD69^−^TCRβ^neg-lo^ DP cells, which have not undergone positive selection, suggesting that positive selection induces USP8-CHMP5 interaction, thus CHMP5 is deubiquitinated and stabilized, enabling CHMP5 to inhibit oxidation and degradation of Bcl-2, thereby promoting survival of positively selected thymocytes (105).

## MARCH8 in CD4 T cell lineage commitment

DP thymocytes interact with MHCII or MHCI molecules expressed on TECs and DCs during positive selection, determining CD4 or CD8 lineage development. TEC and DC present self-antigens to thymocytes through MHCII, facilitating positive selection and negative selection of CD4^+^ T cells. It was found that the E3 ubiquitin ligase MARCH8 targets and regulates the stability of MHCII in TECs (110, 111). MARCH8 deletion leads to increased MHCII protein levels in cTEC and AIRE^-^ mTEC, while the expression of MHCII in AIRE^+^ mTEC is not affected, suggesting MARCH8 modulates protein stability of MHCII (110, 111). However, the number and TCR repertoire of *March8^-/-^* CD4^+^ T cells in thymus and spleen are comparable to wild-type mice (110, 111). CD83 is expressed by TEC and activated T cell, B cell, and DCs. MARCH8 regulates MHCII stability through antagonizing CD83 for MHCII binding in TECs (112, 113). CD83 deletion in TEC leads to impaired MHCII expression and CD4^+^ T cell development, with significantly reduced CD4^+^ SP cells in thymus, while DP and DN cells are normal (111). Notably, either MHCII^K225R^ mutation (preventing MHCII ubiquitination) or MARCH8 deletion can rescue defects of CD4^+^ T cell development in CD83 deficient mice, suggesting MARCH8 and CD83 modulate MHCII ubiquitination to regulate CD4^+^ T cell development. Additionally, CD83 binds and maintains MHCII protein stability, competing with another E3 ubiquitin ligase MARCH1 for MHCII binding in DCs, thereby antagonizing MARCH1-mediated MHCII ubiquitination (114, 115). In contrast, MARCH1 does not affect MHCII expression in TEC. In summary, MARCH8 antagonizes CD83 to control MHCII ubiquitination and stability in TEC, thereby modulating CD4^+^ T lineage commitment (110, 111).

## LUBAC in maturation of late thymocytes

The linear ubiquitin chain assembly complex (LUBAC) is composed of at least 3 proteins: RING-containing E3 ubiquitin ligase (RNF31, also called HOIP), RANBP2-type, C3HC4-type zinc finger-containing 1 (RBCK1, also called HOIL-1L) and SHANK-associated RH domain interactor (SHARPIN, also called SIPL1). LUBAC catalyzes linear ubiquitin chains of substrate proteins, thereby regulating various signaling pathways (116). The three components ensure optimal LUBAC catalytic activity with different extents. HOIP deletion is embryonically lethal and completely abrogates E3 activity of LUBAC (117). Cells lacking SHARPIN still retain substantial linear ubiquitination, as the HOIP-HOIL-1L complex is sufficient to maintain LUBAC E3 catalytic capability (118). Patients with loss-of-function mutations of HOIL-1L and HOIP have reduced T cells, suggesting HOIL and HOIP are involved in T cell homeostasis or development (119). T cell specific knockout of *Rnf31* or *Rbck1* genes (*Rnf31^ΔCD4^* or *Rbck1^ΔCD4^* mice) by *Cd4-cre* leads to nearly complete loss of CD4^+^ and CD8^+^ αβT cells in peripheral lymphoid organs (120). The proportion of Tregs among CD4^+^ αβT cells appears normal but the absolute number is significantly reduced (120). The remaining CD4^+^ and CD8^+^ αβT cells are mostly CD44^hi^CD62L^lo^ phenotype, indicative of an effector/memory phenotype (120). In contrast, *Sharpin* deleted (*Sharpin^cpdm^*) mice have normal CD4^+^ and CD8^+^ T cells, but reduced Treg among CD4^+^ T cells (120). Analysis of thymocyte subsets shows that HOIP and HOIL-1L do not affect the positive and negative selection of early thymocytes, but regulate the maturation of late thymocytes. *Rnf31^ΔCD4^* and *Rbck1^ΔCD4^* mice have reduced CD4^+^ and CD8^+^ SP thymocytes with normal DPs (120). *Sharpin^cpdm^* mice show normal percentages and numbers of DN, DP and SP thymocytes (120). Recent studies suggested two potential pathways of thymic Tregs development involving distinct Treg precursors (TregP): CD25^+^FOXP3^-^ (CD25^+^ TregP) and CD25^-^FOXP3^lo^ (FOXP3lo TregP) (121). CD25^+^ TregP is believed exist at the TCR-instructive phase to generate CD25^+^FOXP3^+^ Tregs following cytokine stimulation in a two-step model of Treg differentiation (122), and is suggested more prone to FOXP3-induced apoptosis (123). Concurrently, both CD25+FOXP3+ Tregs and CD25-FOXP3+ Treg precursors are significantly reduced in the thymuses of *Rnf31^ΔCD4^*, *Rbck1^ΔCD4^* and *Sharpin^cpdm^* mice, indicating HOIP, HOIL-1L and SHARPIN all promote Treg development in the thymus, while HOIP and HOIL-1L additionally regulate CD4^+^ and CD8^+^ T cell development (120).

Further studies revealed that HOIP and HOIL-1L regulate NF-κB activation in thymocytes. Thymocytes lacking HOIP show delayed IκBα degradation upon anti-CD3 and anti-CD28 stimulation. Additionally, Thymocytes lacking HOIP or HOIL-1L exhibit delayed and reduced p65 phosphorylation and IκBα degradation, as well as dampened p38 phosphorylation in MAPK signaling pathway, upon TNF stimulation (120). *Rnf31^ΔCD4^* IKKca mice with sustained IKKβ activation show rescued proportions of mature CD4^+^ SP T cells (CD4^+^CD24^low^CD62L^high^) and FOXP3^+^ Tregs in thymus, while peripheral T cell numbers remains unreplenished (120), suggesting LUBAC impacts thymocyte development via NF-κB pathway as well as other mechanisms. Furthermore, constitutive activation of IKKβ promotes the development of Tregs by facilitating the binding of c-Rel to FOXP3 enhancer region, leading to the transcriptional induction of FOXP3 (124).

## Ubiquitination regulation of thymocytes proliferation and apoptosis

Thymocyte development relies on signals induced by both TCR and cytokine receptors. During thymocyte development, the sequential selections induce several rounds of proliferation and apoptosis, ensuring the diversification of TCR repertoire and elimination of self-reactive T cells. Thymocytes undergo two main rounds of proliferation during DN stage, one before TCRβ rearrangement mediated by stem cell factors (SCF), IL-7 and NOTCH. Mice with IL-7 or IL-7R deficiency are defective in thymocyte proliferation and DN2/3 cell number (125–129). Another is during β-selection when DN3 cells with successful β chain rearrangement express pre-TCR and massively expand (126, 130–132). Cell cycle progression from G0 to G1 and S/G1/M active phases during DN proliferation is controlled by Cyclin-dependent kinases (cyclin-CDK) complexes (133). Cyclin-CDK activity and cell cycle progression are inhibited by the Cip/Kip family including Cdkn1a, Cdkn1b and Cdkn1c, among which Cdkn1b plays a major role in β-selection (134–136). Cdkn1b mainly inhibits the cyclin A-CDK and cyclin E-CDK complexes. *Cdkn1b*-knockout mice have enlarged thymuses (134, 135), while Cdkn1b overexpression causes small thymuses with blocking T cell development at DN3 stage (137).

## FBXL1 and FBXL12 in DN cells proliferation

FBXL1 and FBXL12, as the substrate recognition subunits of Skp-cullin-F-box (SCF) E3 ubiquitin ligases, regulate DN thymocyte proliferation (138–140). In thymocytes, NOTCH signaling induces FBXL1 expression while pre-TCR induces FBXL12 expression (140, 141). SCF complexes containing FBXL1 and FBXL12 collaboratively mediate K48-linked ubiquitination and proteasomal degradation of Cdkn1b, thereby promoting proliferation of DN cells after β-selection (140, 142, 143). *Fbxl1*-knockout mice have significantly smaller thymus, with normal DN1 and DN2 cells but severely impaired DN3 to DN4 development and reduced proliferative capacity of DN4, ISP and DP cells (140). Concurrent *Cdkn1b* deletion restores normal thymus size in *Fbxl1*-knockout mice (144). Germline deletion of *Fbxl12* is embryonic lethal (145). *Lck-cre* mediated conditional *Fbxl12*-deletion results in a phenotype similar to that in *Fbxl1* ^-/-^ mice (140). *Lck-cre* ^+^ *Fbxl12^fl/fl^* mice have increased Cdkn1b protein level in total DN thymocytes, and *Cdkn1b* deletion also rescues the defects in DN3-DN4 development caused by FBXL12 deficiency (140). Additionally, combined FBXL12 and FBXL1 knockout further impairs DN4, ISP and DP proliferative capacity, indicating the collaborative roles of FBXL12 and FBXL1 (140). In summary, FBXL1 and FBXL12 relieve Cdkn1b inhibition on proliferation of DN cell, thereby promoting β-selection-associated proliferation through SCF-mediated Cdkn1b ubiquitination and proteasomal degradation (140).

## USP8 in thymocyte proliferation

The ubiquitin-specific protease 8 (USP8) belongs to USP deubiquitinase family. *Cd4-cre* mediated T cell specific knockout of *Usp8* (*Usp8*-TKO) results in significantly reduced percentages and numbers of CD4^+^ and CD8^+^ SP thymocytes, with unchanged number of DPs (146). The proportion of CD25^+^FOXP3^+^ Tregs among CD4^+^ thymocytes is also markedly reduced (146). Researchers also found TCRβ the expression of CD4^+^ and CD8^+^ SP thymocytes is nearly absent in *Usp8*-TKO mice (146). Further studies show USP8 regulates thymocyte proliferation without affecting negative selection. T cells lacking *Usp8* fail to proliferate upon stimulation *in vitro*. In *Usp8*-knockout DP cells, mRNA levels of *Il7ra* and *Ccr7* are reduced (146), and FOXO1 binding to *Il7ra* promoter is significantly decreased in *Usp8*-knockout CD4^+^ thymocytes, which regulates expression of *Il7ra*, *Ccr7* and *Sell (* 147, 148). Similar to mice with T cell specific *Il7ra* knockout, *Usp8*-TKO mice have a greater reduction of CD8^+^ SP thymocytes in the TCR^hi^ subset, suggesting USP8 regulates T cell development through FOXO1-IL7Rα axis (146). However, the binding of FOXO1 to the *Il7ra* promoter by USP8 is not mediated thorough ubiquitination directly. In detail, USP8 is shown to interact with the adaptor Gads and the regulatory molecule 14-3-3β. 14-3-3β belongs to the 14-3-3 family, which regulates the localization, DNA binding, and transcriptional activity of FOXO1 (149). By co-expression of USP8, 14-3-3β, and ubiquitin, ubiquitination of 14-3-3β at Lys9 and Lys 189 by mass spectrometry is identified (146). In this regard, USP8 might regulate the binding of FOXO1 to DNA indirectly by the regulating the ubiquitination of 14-3-3β. However, there is no direct ubiquitination experiment to reveal that USP8 mediates deubiquitination of 14-3-3β. Otherwise, USP8 binds Gads via its SH3BM domain, and ectopic re-expression of SH3BM-laking mutant of USP8 do not restore T cell development. Moreover, the catalytic activity of USP8 is required for T cell development, as demonstrated by *in vivo* rescue experiment (146). Additionally, deletion of *Usp8* also causes reduced CD4^+^ SP thymocytes. These findings suggest that USP8 regulates T cell development through both FOXO1-IL-7Rα-dependent and independent mechanisms.

## pVHL in apoptosis of thymocyte

The von Hippel-Lindau protein (pVHL) is the substrate recognition component of E3 ubiquitin ligase complex that targets protein for degradation. pVHL is well known as a tumor suppressor through regulating the proteasomal degradation of hypoxia-inducible factor alpha (HIF-α) under normal oxygen conditions (150, 151). pVHL dysfunction leads to VHL disease, a genetic disorder characterized by increased cancer risk (152). Inactivation of pVHL in mouse germ line is embryonic lethality. Using mice with thymocyte-specific *Vhlh* (encoding VHL) deletion by *Lck-cre*, it is revealed that pVHL regulates thymocyte apoptosis. Thymi of *Vhlh*-deficient mice is small and highly vascularized, and the number of DP thymocytes is severely reduce (153). Further study reveals decreased expression of BCL-X_L_ and increased caspase 8 activity in *Vhlh*-deficient thymocytes, which results in impaired survival and increased apoptosis (153). pVHL regulates the ubiquitination and degradation of HIF-α subunits, which has been shown to promote apoptosis (154). Conditional deletion of HIF-1α in pVHL deficient thymocytes restores the expression of BCL-X_L_ and caspase 8 activity in DPs (153). Furthermore, it is demonstrated that HIF-1-dependent caspase 8 activity results in increased apoptosis of thymocytes but not BCL-X_L_ through transgenic expression of BCL-X_L_ and caspase 8-specific inhibitor assays, suggesting the HIF-1-dependent caspase8 activity is required for thymocyte development and viability regulated by pVHL (153).

## Regulatory T cell development

FOXP3-expressing regulatory T (Treg) cells have two major roles in immune system: the prevention of autoimmunity and maintenance of immune homeostasis (155, 156). Based on origin and mechanism of functions, Tregs are divided into tTregs (thymus-derived Tregs) and pTregs (peripherally derived Tregs). tTregs recognize self-antigens presented by MHCII and develop in thymic medulla (157). FOXP3 and IL-2 are critical for the development and maintenance of tTregs. Mature tTregs migrate to the periphery, and function in immune tolerance (158–160). pTregs differentiate from peripheral CD4^+^ T cells under the induction of TGF-β, and mainly secrete IL-10 and TGF-β, playing immune suppression functions at inflammatory sites (161, 162).

Although most self-reactive CD4^+^ T cells are eliminated in the thymic medulla, some self-reactive CD4 T cells can differentiate into Tregs (13, 122, 163, 164). The strength of TCR recognizing self-peptide–MHC complexes is the key factor determining the cell fate of thymocyte. Low-affinity TCR interactions are required for positive selection, while high-affinity TCR interactions lead to negative selection and T cell apoptosis (165). However, some T cell with high-affinity TCRs for self-antigens are not eliminated, but instead undergo clonal diversion to Treg cells (157, 166–168). It is widely accepted that within a certain range of TCR affinity for self-antigen, the likelihood of Treg generation increases. This range typically falls between the thresholds for positive and negative selection (85, 169–172). Treating humanized mice with anti-CD28 antibody increases the number of CD25^+^FOXP3^+^ Tregs in thymus (173, 174). Mice lacking CD28 have greatly reduced numbers and proportions of tTregs in thymus and spleen (160). In addition, patients lacking ZAP70 have significantly decreased numbers and proportions of tTregs. Given the critical roles of CD28 and ZAP70 in TCR activation, these results indicate a promoting effect of TCR signaling on Treg differentiation (175).

## Cbl-b in Treg development

Cbl-b plays a key role in the regulation of TCR signaling thresholds. *Cblb* ^-/-^ T cells have enhanced IL-2 production and cell proliferation upon TCR activation (176, 177). It has been shown that Cbl-b is involved in regulating tTreg and pTreg development via CD28 co-stimulation, which induces expression of FOXP3 in DP thymocytes (178). Deficiency of Cbl-b results in increased tTreg. CD28 deficiency leads to dramatically reduced tTreg numbers in both thymus and periphery (160), while Cbl-b deletion can partially restore tTreg development in *Cd28^-/-^* mice. Mechanistic studies found that Cbl-b binds FOXP3 upon TCR activation and, together with STUB1, mediates ubiquitination and subsequent proteasomal degradation of FOXP3. Proteasome inhibition can restore tTreg development in *Cd28^-/-^* mice, similar to the effect of Cbl-b deletion in *Cd28^-/-^* mice, indicating that Cbl-b regulates tTreg development by ubiquitinating and degrading FOXP3 (178).

Cbl-b also regulates pTreg differentiation induced by TGF-β. In contrast to tTreg development, Cbl-b regulates pTreg development by modulating FOXP3 expression instead of degradation, suggesting different mechanisms in the thymus and periphery. Transcription factors Forkhead-box O (FOXO) family proteins FOXO3a and FOXO1 directly bind the promoter of FOXP3 and induce its transcription, and phosphorylation of FOXO3a and FOXO1 prevents its binding to the FOXP3 promoter (179). Cbl-b deficiency leads to reduced TGF-β-induced pTreg cell numbers (179, 180). Mechanism study revealed enhanced phosphorylation of FOXO3a and FOXO1 in Cbl-b-deficient CD4 T cells upon TGF-β stimulation, leading to decreased FOXP3 expression (179). Expressing FOXO3a in Cbl-b-deficient mice can restore expression of FOXP3, indicating that Cbl-b inhibits the phosphorylation of FOXO3a and FOXO1, thereby promoting the expression of FOXP3 and development of pTreg (179). However, how Cbl-b, as an E3 ubiquitin ligase, inhibits phosphorylation of FOXO3a and FOXO1 remains to be elucidated. Another study showed that AKT2 mediates phosphorylation of FOXO3a and FOXO1, and the phosphorylation of AKT is enhanced in Cbl-b-deficient T cells (181). AKT2 deletion eliminates excessive phosphorylation of FOXO3a and FOXO1 caused by Cbl-b deficiency, suggesting that Cbl-b regulates FOXO phosphorylation and subsequent pTreg development through modulating AKT activation, although the underlying mechanisms of how Cbl-b regulates AKT phosphorylation as an E3 ligase requires further investigation (181). Furthermore, recent study suggests that TGFβ signaling promotes Treg differentiation by disrupting weaker agonist TCR signaling to promote nuclear FOXO1 expression (182). Given that Cbl-b-deficient T cells show hyperactivation of AKT upon TCR stimulation (181, 183), it is possible that Cbl-b deficiency enhances T cell activation and therefore overcomes TGFβ-mediated suppression of TCR signaling, thus leading to enhanced AKT activation to enhance the phosphorylation of FOXO3a and FOXO1, and suppressed FOXP3 expression. Additionally, studies show that TGF-β stimulation leads to reduced phosphorylation of downstream SMAD2 and decreased FOXP3^+^ Treg generation in Cbl-b deficient mice, suggesting Cbl-b regulates FOXP3 expression and Treg induction via modulating the TGF-β/SMAD2 signal pathway (180).

## MARCH1 in Treg development

MARCH1 is a RING domain-containing E3 ubiquitin ligase. Treg development in thymus requires antigen presentation by DCs and TECs (184, 185). MHCII-expressing DCs promote negative selection and Treg generation in the thymus (186, 187). MARCH1 regulates ubiquitination of MHCII and CD86 in DCs, leading to internalization, lysosomal targeting, and degradation of the indicated molecules, which dampens DC antigen presentation (186, 188). Upon exposure to maturation signals like microbes and inflammatory stimuli, MARCH1 expression is downregulated and MHCII/CD86 levels increase, enhancing antigen presentation of DCs (189, 190). Under immunosuppressive conditions like IL-10 stimulation, MARCH1 expression is upregulated on DCs, thus decreasing MHCII/CD86 protein levels and antigen presentation capacity (115, 186). By regulating ubiquitination of MHCII in thymic DCs, MARCH1 controls tTreg development (191). MARCH1 deficiency leads to dramatically reduced tTreg numbers without affecting the total CD4^+^ thymocytes, and MARCH1-deficient DCs fail to support antigen-specific Treg development *in vivo* and *in vitro (* 191). MHCII mutant mice that cannot be ubiquitinated also exhibit reduced tTreg numbers (191, 192), highlighting the importance of MARCH1-mediated MHCII ubiquitination in tTreg development. Given the higher-strength signals compared to positive selection, and lower-strength signals compared to negative selection are required for Treg development, it is possible that the impaired Treg development in MARCH1 deficient mice is due to the strong TCR signaling caused by increased MHCII/CD86 and antigen presentation capacity.

## Itch in Treg development

Itch is a HECT-containing E3 ubiquitin ligase that mainly regulates T cell activation (193). *Itch^-/-^* T cells are unresponsive to TGF-β stimulation, leading to impaired FOXP3 expression and reduced Treg differentiation (194). Mechanistic studies found that Itch binds the transcription factor TGF-β inducible early gene 1 (TIEG1) and mediates its mono-ubiquitination and poly-ubiquitination without affecting its protein stability (194). Ubiquitinated TIEG1 directly binds to the FOXP3 promoter and induces FOXP3 transcription. Deficiency of either TIEG1 or Itch dramatically reduces FOXP3 expression and Treg differentiation, suggesting Itch, together with TIEG1, promote the expression of FOXP3 and Treg differentiation (194).

Additionally, other studies show Itch regulates TGF-β-induced Treg differentiation via the adaptor protein Ndfip1 (195). *Itch* ^-/-^ T cells exhibit increased proliferation and preferential Th2 differentiation with elevated production of Th2-associated cytokines like IL-4 and IL-5 (196, 197). It has been studied that Itch binds and mediates ubiquitination and degradation of the transcription factor JunB, thus inhibiting IL-4 production (196–198). The adaptor protein Ndfip1 is critical for Itch-mediated JunB ubiquitination. Upon T cell activation, Ndfip1 associates with Itch and promotes Itch-mediated JunB degradation (199, 200). Deficiency of either Ndfip1 or Itch leads to prolonged JunB half-life and enhanced Th2 responses with elevated IL-4 production (199), and TGF-β-induced FOXP3^+^ Treg generation is impaired (195). Ndfip1 deficiency does not affect the binding of TIEG1 to the FOXP3 promoter, indicating Ndfip1/Itch regulate FOXP3^+^ Treg differentiation independently of TIEG1. Furthermore, subsequent studies show that increased IL-4 production in *Ndfip1* ^-/-^ cells inhibits FOXP3 expression (195, 201). Therefore, it is proposed that Ndfip1 facilitates Itch-mediated ubiquitination and degradation of JunB, inhibiting IL-4 production and promoting FOXP3^+^ Treg differentiation (195).

## TRAF3 in Treg development

TRAF3, a member of the tumor necrosis factor receptor-associated factor family, mainly participates in TNF receptor (TNFR) and Toll-like receptor (TLR) signaling pathway. TRAF3 contains TRAF domains and a RING domain, enabling its function as an adaptor protein to facilitate signaling transduction or E3 ubiquitin ligase to mediate ubiquitination (202). It is shown that TRAF3 negatively regulates tTreg development in thymus by modulating Treg precursor differentiation, a process requiring IL-2 signaling (122, 203, 204). IL-2 activates downstream pathways upon binding to IL-2 receptor (IL-2R), which is consisted of tree subunits, IL-2Rα (CD25), IL-2Rβ (CD122), and common γ chain (CD132) (205). JAK1 and JAK3 associate with CD122 and CD132, respectively, and mediate phosphorylation of themselves as well as IL-2Rβ and the transcription factor STAT5 (206, 207). Phosphorylated STAT5 binds to the FOXP3 promoter and induces FOXP3 expression (208, 209). TRAF3 recruits the tyrosine phosphatase TCPTP to the IL-2R complex, leading to dephosphorylation and inactivation of JAK1/3 and subsequent STAT5 signaling, thus inhibiting tTreg development (203). However, whether TRAF3 functions as an E3 ligase or adaptor in this process remains unclear. Additionally, Treg-specific TRAF3 deletion impairs Treg function, disrupts CD4 T cell homeostasis, increases Th1-like effector/memory T cells, and enhances Tfh activation and germinal center formation as well as IgG production (210). Another TRAF molecule, TRAF6, has also been reported to regulate Treg function by mediating K63-linked ubiquitination of FOXP3, ensuring nuclear localization of FOXP3 for its transcriptional activity. *Traf6*-deficient Tregs showed perinuclear accumulation of FOXP3, resulting in impaired immunosuppressive capacity and enhanced anti-tumor immunity (211).

## A20 inhibits Treg development

A20 is a deubiquitinase that mainly mediates negative feedback regulation of NF-κB signaling and regulates TNF-induced apoptosis (212). A20 plays important roles in T cell activation, survival, and differentiation. Upon TCR activation, A20 removes ubiquitination of MALT1, thus inhibiting NF-κB activation (213, 214). A20 also inhibits receptor interacting protein kinase 3 (RIPK3)-induced necroptosis of naïve CD4 T cells (215). It was shown that the number of Treg cells is significantly increased in both the thymus and periphery in mice with T cell-specific A20 deletion (216). A20-deficient Tregs are less dependent on IL-2 with unaffected proliferation and apoptosis. Thymic Treg cell progenitors (CD4^+^CD25^+^GITR^+^FOXP3^-^) are also markedly increased in mice with T cell-specific A20 deletion, along with increased GITR expression, which is a member of the tumor necrosis factor receptor superfamily (TNFRSF) protein and promotes Treg development (216). TNFRSF agonists enhance activation of IL-2R and STAT5, thus promoting the differentiation of Treg cell progenitors (217). GITR deficiency impairs Treg development, and combined inhibition of GITR, OX40, and TNFR2 abrogates the development of tTreg (217), highlighting the role of GITR in promoting tTreg development. Therefore, increased GITR expression in T cell-specific A20-deficient mice promotes tTreg development.

## CYLD inhibits Treg development

The deubiquitinase CYLD regulates both CD4^+^/CD8^+^ T cell and Treg development, although different CYLD deletions have differential effects. Mice lacking exons 2 and 3 of *Cyld* gene exhibit significantly increased peripheral and normal thymic Treg numbers (218). *In vitro*, TGF-β-induced differentiation of Tregs from naïve CD4^+^ T cells is enhanced upon *Cyld* deletion (exons 2 and 3) (218). Mechanistic studies found that CYLD binds SMAD7 and removes ubiquitination chains at Lys360/Lys374 sites of SMAD7, thus dampening the activation of TAK1, p38, as well as the transcription factor AP-1 (218). SMAD7 or p38 deficiency inhibits Treg differentiation, indicating that CYLD regulates TGF-β signaling and inhibits Treg differentiation by deubiquitinating SMAD7 and dampening AP-1 activation (218).

Lacking of exons 7 and 8 of *Cyld* (*Cyld^ex7/8^*) abrogates the interaction of CYLD-NEMO and CYLD-TRAF2 (219). *Cyld^ex7/8^* mice, exclusively express a naturally occurring splice variant of CYLD lacking exons 7 and 8, exhibit constitutive NF-κB activation in T cells and produce higher levels of proinflammatory cytokines like IL-17A, IL-6, and IFN-γ (220). Concurrently, tTreg and pTreg numbers are significantly increased in *Cyld^ex7/8^* mice. However, these Tregs exhibit reduced CD25 and CTLA-4 expression associated with impaired suppressive capacity, indicating that while CYLD inhibits tTreg development, it promotes Treg regulatory function to control abnormal T cell responses (220).

## mTEC and establishment of self-tolerance

Positive and negative selection are highly regulated processes dependent on the distinct thymic microenvironment composed of stromal cells including thymic epithelial cells (TECs), endothelial cells, mesenchymal cells, and non-lymphoid cells like dendritic cells (DCs) and macrophages (221, 222). Among them, TECs play critical roles. TECs exert important functions in selection of αβT cells, Treg generation, and development of other unconventional T cell subsets (223, 224). TECs provide migration, proliferative, differentiative and survival signals for developing thymocytes to shape a diverse and functional T cell repertoire without self-reactivity, thus establishing central tolerance, preventing peripheral autoimmune responses (225, 226). Based on anatomical localization, marker expression, and functional differences, TECs are classified into cortical TECs (cTECs) and mTECs (225, 227)。 cTEC resides in the thymic cortex region and expresses various molecules critical for early T cell development and positive selection, such as IL-7, MHC, the NOTCH ligands DLL1 and DLL4, proteases β5t, TSSP and CTSL, to constitute the unique cortical microenvironment supporting DN and DP cell development (85, 225, 228). mTEC localizes in the medulla region and plays vital roles in negative selection and self-tolerance establishment (229, 230).

mTECs promiscuously express tissue-restricted antigens (TRAs) to mediate removal of thymocytes bearing self-reactive TCRs, establishing central tolerance to avoid autoimmune attack of self-antigens by T cells exiting to the periphery (231, 232). The transcription factor autoimmune regulator (AIRE) is crucial for TRA expression of mTEC. Deficiency or loss-function mutations of AIRE reduce TRA expression in mice, leading to defective central tolerance and escape of autoreactive T cells (233–236). Loss-function mutations of AIRE in human also result in multiple organ-specific autoimmune diseases, such as autoimmune polyendocrinopathy-candidiasis-ectodermal dystrophy (APECED), otherwise known as autoimmune polyendocrine syndrome type 1 (APS-1) (237, 238). AIRE contains two plant homeodomains (PHDs) with similar structure to RING domains (239, 240). Some studies suggest the PHD1 domain possesses E3 ubiquitin ligase activity that impacts AIRE function in central tolerance (241), but whether AIRE functions as an E3 ligase remains controversial as other studies found no ubiquitin ligase activity (242).

## TRAF6 promotes mTEC development

As an E3 ubiquitin ligase, TRAF6 activates classical NF-κB downstream of TLR and TCR signaling. TRAF6 deficiency causes thymic atrophy, indicating its critical role in promoting organogenesis (243). Further studies found that TRAF6 deletion disrupts mTEC distribution in the thymus, reduces mature mTECs, and impairs thymic architecture (244). Additionally, transplantation of thymic stroma of *Traf6*-deficient embryos into athymic nude mice causes inflammatory response in lung, liver, pancreas, and kidney, as well as splenomegaly along with high levels of autoantibodies against pancreas and lung (244), indicating TRAF6 deficiency leads to autoimmunity.

NF-κB is activated through canonical and non-canonical pathways. Substantial evidence demonstrates the role of non-canonical NF-κB signaling pathway in promoting mTEC development. Deficiency of the key proteins in non-canonical NF-κB activation like RANK, CD40, LIGHT, and LTβR in mice impairs both mTEC development and function, leading to autoimmune symptoms (224, 226, 243). As the transcription factor of non-canonical NF-κB signaling, RelB deficiency reduces expression of AIRE and functional mTECs, which results in multi-organ inflammatory response (245–247), highlighting the importance of non-canonical NF-κB in mTEC development. FTOC experiments using TRAF6-deficient embryos found that RANKL and CD40L stimulation fails to induce UEA^+^Aire^+^ mTEC and inhibits AIRE and TRA expression, indicating the vital role of TRAF6 in mTEC development and function (248). Further studies revealed that RelB expression is significantly reduced in medullary cells of TRAF6-deficient thymus, and RANKL/CD40L stimulation of TRAF6-deficient thymic organ cultures cannot induce RelB expression either, suggesting TRAF6 regulates mTEC development by modulating RelB expression (244, 248).

TEC-specific TRAF6 deletion reveals normal cTEC development but dramatically reduced mature and immature mTECs (249). Additionally, TEC-specific TRAF6 knockout mice have reduced body weight, inflammatory response in the liver, lungs, and kidneys, and exhibit autoantibodies against the liver, lungs, kidneys, small intestine, and colon, while features of human autoimmune hepatitis (AIH) including increased plasmacytes and liver-reactive T cells are also observed in the livers (249). Transferring liver T cells into immunodeficient mice recapitulates AIH symptoms, confirming that TRAF6 directly regulates mTEC development to impact self-reactive T cells and autoimmunity (249). However, the detailed molecular mechanisms of how TRAF6, as an E3 ubiquitin ligase, regulates RelB expression and mTEC development remain unclear. Given the role of TRAF6 in classical NF-κB signaling, it is proposed that TRAF6 may modulate RelB expression and mTEC development via activating the classical NF-κB pathway (248).

## TRAF3 in mTEC development

mTEC promotes the induction of self-tolerance in developing T cells. In turn, development of mTEC requires signals from the developing thymocytes, a process termed crosstalk (250–252). As a negative regulator of non-canonical NF-κB pathway, TRAF3, together with TRAF2, facilitates the ubiquitination and degradation of NIK to maintain extremely low NIK levels. In *Tcra^-/-^* mice, T cell development is arrested at the DP stage, with a complete absence of SP thymocytes as well as significantly decreased mTEC regions (253). TRAF3 was reported to enforce the requirement for T cell crosstalk in mTEC development (254). TEC-specific deletion of TRAF3 using *Foxn1-cre* mice in *Tcra^-/-^* mice restores the development of mTEC, with confluent medullary areas and normal AIRE expression (254). Furthermore, TEC-specific deletion of TRAF3 in *Ltbr^-/-^/Ltbr^-/-^* double-deficient mice restores the defects in mTEC development (254). These results suggest TRAF3 deletion can overcome requirement for thymocyte crosstalk as well as LTβR and CD40 signals. However, TEC-specific TRAF3 deletion fails to rescue mTEC development in *Relb^-/-^* mice, suggesting its role is RelB dependent (254). Given the critical role of non-canonical NF-κB pathway in mTEC development, it is likely TRAF3 regulates mTE development by facilitating the ubiquitination and degradation of NIK.

## CYLD promotes mTEC development

Besides its roles in thymic positive selection and Treg development mentioned above, CYLD also regulates mTEC development and function, thereby impacting thymic negative selection (255). In *Cyld^ex7/8^* mice, development from immature mTEC to mature mTECs is blocked, and mature mTECs are dramatically reduced. Regarding the role of CYLD in T cell development, CYLD^FL^-CD4-cre mice are utilized, in which sCYLD (lacking exons 7 and 8) is overexpressed solely in αβT cells. The results show normal mTEC numbers, indicating that CYLD directly regulates mTEC development independent of effects on T cells. However, whether the development of thymocyte was affected in CYLD^FL^-CD4-cre mice has not been shown. Furthermore, to assess the impact of CYLD deletion on negative selection, HYtg mice were crossed with *Cyld^ex7/8^* mice and the results show that HYtg *Cyld^ex7/8^* male mice contain detectable DP, CD4^+^ SP, and CD8^+^ SP thymocytes, indicating impaired negative selection and escape of autoreactive T cells upon CYLD deletion (exons 7/8). This demonstrates that CYLD regulates mTEC development and maturation to impact negative selection and establishment of central tolerance (255).

## LUBAC promotes TEC survival

The role of LUBAC in thymocyte development has been discussed above. TEC-specific knockout of HOIL-1L or HOIP also affects the development and survival of TEC, thereby affecting T cell development in the thymus (256). Using *Foxn1-cre* mice to delete *Rbck1* (*Hoil1^ΔFoxn1^*) or *Rnf31* (*Hoip^ΔFoxn1^*) specifically in TEC, it is found that the thymus of adult mice (8 weeks old) is severely atrophied, and DN, DP and SP cells are significantly reduced (256). The absolute numbers of DN1, DN2, DN3 and DN4 thymocytes are significantly reduced, among which the proportion of DN3 is decreased, suggesting the development of DN3 thymocytes is blocked (256). Additionally, TEC is significantly reduced in adult *Hoil1^ΔFoxn1^* and *Hoip^ΔFoxn1^* mice (256). Immunofluorescence staining shows structural defects in K8^+^ cortical area and K5^+^ or UEA-1^+^ medullary area of *Hoil1^ΔFoxn1^* (or *Hoip^ΔFoxn1^*) thymus (256). AIRE^+^ mTECs are almost completely disappeared, while ERTR7^+^ fibroblasts are prominently remodeled (256). mTECs including AIRE^+^ mTEC are also significantly decreased in embryonic and newborn *Hoil1^ΔFoxn1^* mice, revealing the dual roles of LUBAC complex in TEC differentiation and survival (256).

Further RNA sequencing reveals that the expression of programmed cell death-related gene is changed in HOIL-1L knockout TECs, among which *Mlkl* and *Casp8* are upregulated in cTEC and mTEC^hi^ (MHCII^hi^CD80^hi^) populations (256). Deletion of *Mlkl* and *Casp8* genes in *Hoil-1^ΔFoxn1^* mice revealed that the total number of thymocytes, DN, DP and SP cells recovers to normal levels, but the number of cTEC and mTEC only partially recovers (256). Immunofluorescence staining results show that the cortical structure is basically restored, but the medullary structure is only partially repaired, suggesting that HOIL-1L affects cTEC survival by regulating Caspase-8/MLKL-mediated cell apoptosis, while there are still other mechanisms regulating mTEC development (256). Otherwise, since *Mlkl* and *Casp8* are germline deleted, the effects reflected in thymocyte development in *Hoil-1^ΔFoxn1^* mice may be caused through thymocyte-intrinsic way (256). Therefore, how LUBAC affects AIRE^+^ mTEC development and whether it affects the establishment of self-tolerance needs more research.

## Concluding remarks

T cell plays critical role in adaptive immunity. Progenitors from the bone marrow enter the thymus and undergo TCR rearrangement, β-selection, positive selection, negative selection, and finally differentiated to mature T cells entering periphery, forming an abundant TCR repertoire that enables the recognition of diverse antigens (155, 257–260). Ubiquitination modulates thymocyte development through regulating several key events including T precursor homeostasis, cell proliferation and apoptosis, pre-TCR and TCR activation to ensure the optimal signals for normal T cell development. Meanwhile, several E3 ubiquitin ligases and DUBs regulate Treg development by modulating critical signals like IL-2 or TGF-β. Negative selection of thymocytes ensures mature T cells enter the periphery without attacking self-tissues, thereby establishing central tolerance, while mTEC plays an indispensable role in this process. E3 ubiquitin ligases or DUBs affect mTEC development and function mainly by regulating the NF-κB pathway, thereby modulating negative selection of T cells, ensuring the establishment of central tolerance.

Impaired negative selection leads to self-reactive T cells entering the periphery, leading to autoimmunity in multiple organs. Therefore, figuring out the regulatory mechanisms is meaningful for normal T cell development and guiding the treatment of autoimmune diseases. Besides role in T cell activation, regulatory role of ubiquitination in T cell development and self-tolerance establishment is relatively less studied. Previous studies mainly focus on the role of single E3 or DUB molecules, while the systematic mechanism underlying how ubiquitination regulates T cell development is absent. Ubiquitinomics advancements make it possible and resolve these questions, which benefits the therapeutic target development, providing novel therapeutic opportunity for autoimmune diseases and cancers.

## Author contributions

HY: Writing – original draft, Writing – review & editing, Conceptualization, Investigation, Software. WY: Conceptualization, Funding acquisition, Writing – original draft. MC: Conceptualization, Funding acquisition, Writing – original draft. QL: Writing – review & editing, Validation. RY: Conceptualization, Formal analysis, Investigation, Writing – review & editing. HX: Conceptualization, Data curation, Investigation, Writing – review & editing. YC: Writing – review & editing, Validation. XC: Writing – review & editing, Validation. XS: Writing – review & editing, Validation. HZ: Conceptualization, Data curation, Funding acquisition, Investigation, Writing – review & editing. LL: Conceptualization, Data curation, Investigation, Writing – original draft, Writing – review & editing.

## References

[1] Hu H Sun SC. Ubiquitin signaling in immune responses. Cell Res. (2016) 26:457–83. doi: 10.1038/cr.2016.40 PMC4822134 27012466

[2] Hershko A Ciechanover A. The ubiquitin system. Annu Rev Biochem. (1998) 67:425–79. doi: 10.1146/annurev.biochem.67.1.425 9759494

[3] Park Y Jin HS Aki D Lee J Liu YC. The ubiquitin system in immune regulation. Adv Immunol. (2014) 124:17–66. doi: 10.1016/B978-0-12-800147-9.00002-9 25175772

[4] Pickart CM. Mechanisms underlying ubiquitination. Annu Rev Biochem. (2001) 70:503–33. doi: 10.1146/annurev.biochem.70.1.503 11395416

[5] Berndsen CE Wolberger C. New insights into ubiquitin E3 ligase mechanism. Nat Struct Mol Biol. (2014) 21:301–7. doi: 10.1038/nsmb.2780 24699078

[6] Husnjak K Dikic I. Ubiquitin-binding proteins: decoders of ubiquitin-mediated cellular functions. Annu Rev Biochem. (2012) 81:291–322. doi: 10.1146/annurev-biochem-051810-094654 22482907

[7] Sun SC. Deubiquitylation and regulation of the immune response. Nat Rev Immunol. (2008) 8:501–11. doi: 10.1038/nri2337 PMC5763493 18535581

[8] Sluimer J Distel B. Regulating the human HECT E3 ligases. Cell Mol Life Sci. (2018) 75:3121–41. doi: 10.1007/s00018-018-2848-2 PMC6063350 29858610

[9] Pao KC Wood NT Knebel A Rafie K Stanley M Mabbitt PD. Activity-based E3 ligase profiling uncovers an E3 ligase with esterification activity. Nature. (2018) 556:381–5. doi: 10.1038/s41586-018-0026-1 29643511

[10] Deshaies RJ Joazeiro CA. RING domain E3 ubiquitin ligases. Annu Rev Biochem. (2009) 78:399–434. doi: 10.1146/annurev.biochem.78.101807.093809 19489725

[11] Dye BT Schulman BA. Structural mechanisms underlying posttranslational modification by ubiquitin-like proteins. Annu Rev Biophys Biomol Struct. (2007) 36:131–50. doi: 10.1146/annurev.biophys.36.040306.132820 17477837

[12] Farber DL. Form and function for T cells in health and disease. Nat Rev Immunol. (2020) 20:83–4. doi: 10.1038/s41577-019-0267-8 31889162

[13] Kumar BV Connors TJ Farber DL. Human T cell development, localization, and function throughout life. Immunity. (2018) 48:202–13. doi: 10.1016/j.immuni.2018.01.007 PMC5826622 29466753

[14] Awong G Zúñiga-Pflücker JC. Module in biomedical sciences. Elsevier (2014). vol. 12. p. 229–39.

[15] Six EM Bonhomme D Monteiro M Beldjord K Jurkowska M Cordier-Garcia C. A human postnatal lymphoid progenitor capable of circulating and seeding the thymus. J Exp Med. (2007) 204:3085–93. doi: 10.1084/jem.20071003 PMC2150974 18070935

[16] Hosokawa HA-O Rothenberg E. A.-O. X. How transcription factors drive choice of the T cell fate. Nature Rev Immunol. (2021) 21(3):162–76. doi: 10.1038/s41577-020-00426-6 PMC7933071 32918063

[17] Res P Spits H. Developmental stages in the human thymus. Semin Immunol. (1999) 11(1):39–46. doi: 10.1006/smim.1998.0152 9952357

[18] Yui MA Rothenberg EV. Developmental gene networks: a triathlon on the course to T cell identity. Nat Rev Immunol. (2014) 14:529–45. doi: 10.1038/nri3702 PMC4153685 25060579

[19] Gascoigne NR Rybakin V Acuto O Brzostek J. TCR signal strength and T cell development. Annu Rev Cell Dev Biol. (2016) 32:327–48. doi: 10.1146/annurev-cellbio-111315-125324 27712102

[20] Fu G Casas J Rigaud S Rybakin V Lambolez F Brzostek J. Themis sets the signal threshold for positive and negative selection in T-cell development. Nature. (2013) 504:441–5. doi: 10.1038/nature12718 PMC3977001 24226767

[21] Oh-Hora M Komatsu N Pishyareh M Feske S Hori S Taniguchi M. Agonist-selected T cell development requires strong T cell receptor signaling and store-operated calcium entry. Immunity. (2013) 38:881–95. doi: 10.1016/j.immuni.2013.02.008 PMC3669219 23499491

[22] Pellicci DG Koay HF Berzins SP. Thymic development of unconventional T cells: how NKT cells, MAIT cells and γδ T cells emerge. Nat Rev Immunol. (2020) 20:756–70. doi: 10.1038/s41577-020-0345-y 32581346

[23] Li X Pu W Zheng Q Ai M Chen S Peng Y. Proteolysis-targeting chimeras (PROTACs) in cancer therapy. Mol Cancer. (2022) 21:99. doi: 10.1186/s12943-021-01434-3 35410300 PMC8996410

[24] Narayanan S Cai CY Assaraf YG Guo HQ Cui Q Wei L. Targeting the ubiquitin-proteasome pathway to overcome anti-cancer drug resistance. Drug resistance updates Rev commentaries antimicrobial Anticancer chemotherapy. (2020) 48:100663. doi: 10.1016/j.drup.2019.100663 31785545

[25] Spano D Catara G. Targeting the ubiquitin-proteasome system and recent advances in cancer therapy. Cells. (2023) 13. doi: 10.3390/cells13010029 PMC10778545 38201233

[26] Galy A Verma S Bárcena A Spits H. Precursors of CD3+CD4+CD8+ cells in the human thymus are defined by expression of CD34. Delineation of early events in human thymic development. J Exp Med. (1993) 178:391–401. doi: 10.1084/jem.178.2.391 7688021 PMC2191105

[27] Hao QL George AA Zhu J Barsky L Zielinska E Wang X. Human intrathymic lineage commitment is marked by differential CD7 expression: identification of CD7- lympho-myeloid thymic progenitors. Blood. (2008) 111:1318–26. doi: 10.1182/blood-2007-08-106294 PMC2214748 17959857

[28] Casero D Sandoval S Seet CS Scholes J Zhu Y Ha VL. Long non-coding RNA profiling of human lymphoid progenitor cells reveals transcriptional divergence of B cell and T cell lineages. Nat Immunol. (2015) 16:1282–91. doi: 10.1038/ni.3299 PMC4653072 26502406

[29] Plum J De Smedt M Leclercq G Taghon T Kerre T Vandekerckhove B. Human intrathymic development: a selective approach. Semin immunopathology. (2008) 30:411–23. doi: 10.1007/s00281-008-0135-2 18925396

[30] Weerkamp F Baert MRM Brugman MH Dik WA de Haas EFE Visser TP. Human thymus contains multipotent progenitors with T/B lymphoid, myeloid, and erythroid lineage potential. Blood. (2006) 107:3131–7. doi: 10.1182/blood-2005-08-3412 16384926

[31] Staal FJ Wiekmeijer AS Brugman MH Pike-Overzet K. The functional relationship between hematopoietic stem cells and developing T lymphocytes. Ann New York Acad Sci. (2016) 1370:36–44. doi: 10.1111/nyas.12995 26773328

[32] Haddad R Guimiot F Six E Jourquin F Setterblad N Kahn E. Dynamics of thymus-colonizing cells during human development. Immunity. (2006) 24:217–30. doi: 10.1016/j.immuni.2006.01.008 16473833

[33] Le J Park JE Ha VL Luong A Branciamore S Rodin AS. Single-cell RNA-seq mapping of human thymopoiesis reveals lineage specification trajectories and a commitment spectrum in T cell development. Immunity. (2020) 52:1105–1118 e1109. doi: 10.1016/j.immuni.2020.05.010 32553173 PMC7388724

[34] Canté-Barrett K Mendes RD Li Y Vroegindeweij E Pike-Overzet K Wabeke T. Loss of CD44(dim) expression from early progenitor cells marks T-cell lineage commitment in the human thymus. Front Immunol. (2017) 8:32. doi: 10.3389/fimmu.2017.00032 28163708 PMC5247458

[35] Dik WA Pike-Overzet K Weerkamp F de Ridder D de Haas EF Baert MR. New insights on human T cell development by quantitative T cell receptor gene rearrangement studies and gene expression profiling. J Exp Med. (2005) 201:1715–23. doi: 10.1084/jem.20042524 PMC2213269 15928199

[36] Germain RN. T-cell development and the CD4-CD8 lineage decision. Nat Rev Immunol. (2002) 2:309–22. doi: 10.1038/nri798 12033737

[37] Krueger A Ziętara N Łyszkiewicz M. T cell development by the numbers. Trends Immunol. (2017) 38:128–39. doi: 10.1016/j.it.2016.10.007 27842955

[38] Seo W Taniuchi I. Transcriptional regulation of early T-cell development in the thymus. Eur J Immunol. (2016) 46:531–8. doi: 10.1002/eji.201545821 26763078

[39] Godfrey DI Kennedy J Suda T Zlotnik A. A developmental pathway involving four phenotypically and functionally distinct subsets of CD3-CD4-CD8- triple-negative adult mouse thymocytes defined by CD44 and CD25 expression. J Immunol (Baltimore Md. 1950). (1993) 150:4244–52. doi: 10.4049/jimmunol.150.10.4244 8387091

[40] Ceredig R Rolink T. A positive look at double-negative thymocytes. Nat Rev Immunol. (2002) 2:888–97. doi: 10.1038/nri937 12415312

[41] Lu M Tayu R Ikawa T Masuda K Matsumoto I Mugishima H. The earliest thymic progenitors in adults are restricted to T, NK, and dendritic cell lineage and have a potential to form more diverse TCRbeta chains than fetal progenitors. J Immunol (Baltimore Md. 1950). (2005) 175:5848–56. doi: 10.4049/jimmunol.175.9.5848 16237077

[42] Shen HQ Lu M Ikawa T Masuda K Ohmura K Minato N. T/NK bipotent progenitors in the thymus retain the potential to generate dendritic cells. J Immunol (Baltimore Md. 1950). (2003) 171:3401–6. doi: 10.4049/jimmunol.171.7.3401 14500634

[43] Masuda K Itoi M Amagai T Minato N Katsura Y Kawamoto H. Thymic anlage is colonized by progenitors restricted to T, NK, and dendritic cell lineages. J Immunol (Baltimore Md. 1950). (2005) 174:2525–32. doi: 10.4049/jimmunol.174.5.2525 15728458

[44] Petrie HT Zúñiga-Pflücker JC. Zoned out: functional mapping of stromal signaling microenvironments in the thymus. Annu Rev Immunol. (2007) 25:649–79. doi: 10.1146/annurev.immunol.23.021704.115715 17291187

[45] Godfrey DI Kennedy J Mombaerts P Tonegawa S Zlotnik A. Onset of TCR-beta gene rearrangement and role of TCR-beta expression during CD3-CD4-CD8- thymocyte differentiation. J Immunol (Baltimore Md. 1950). (1994) 152:4783–92. doi: 10.4049/jimmunol.152.10.4783 7513723

[46] Smeets MF Wiest DL Izon DJ. Fli-1 regulates the DN2 to DN3 thymocyte transition and promotes γδ T-cell commitment by enhancing TCR signal strength. Eur J Immunol. (2014) 44:2617–24. doi: 10.1002/eji.201444442 PMC5242326 24935715

[47] Joachims ML Chain JL Hooker SW Knott-Craig CJ Thompson LF. Human alpha beta and gamma delta thymocyte development: TCR gene rearrangements, intracellular TCR beta expression, and gamma delta developmental potential–differences between men and mice. J Immunol (Baltimore Md. 1950). (2006) 176:1543–52. doi: 10.4049/jimmunol.176.3.1543 PMC1592528 16424183

[48] Carbone M Yang H Pass HI Krausz T Testa JR Gaudino G. BAP1 and cancer. Nat Rev Cancer. (2013) 13:153–9. doi: 10.1038/nrc3459 PMC3792854 23550303

[49] Carbone M Harbour JW Brugarolas J Bononi A Pagano I Dey A. Biological mechanisms and clinical significance of BAP1 mutations in human cancer. Cancer Discovery. (2020) 10:1103–20. doi: 10.1158/2159-8290.cd-19-1220 PMC8006752 32690542

[50] Arenzana TL Lianoglou S Seki A Eidenschenk C Cheung T Seshasayee D. Tumor suppressor BAP1 is essential for thymic development and proliferative responses of T lymphocytes. Sci Immunol. (2018) 3. doi: 10.1126/sciimmunol.aal1953 29678836

[51] Tetzlaff MT Yu W Li M Zhang P Finegold M Mahon K. Defective cardiovascular development and elevated cyclin E and Notch proteins in mice lacking the Fbw7 F-box protein. Proc Natl Acad Sci U.S.A. (2004) 101:3338–45. doi: 10.1073/pnas.0307875101 PMC373463 14766969

[52] Tsunematsu R Nakayama K Oike Y Nishiyama M Ishida N Hatakeyama S. Mouse Fbw7/Sel-10/Cdc4 is required for notch degradation during vascular development. J Biol Chem. (2004) 279:9417–23. doi: 10.1074/jbc.M312337200 14672936

[53] Malyukova A Dohda T von der Lehr N Akhoondi S Corcoran M Heyman M. The tumor suppressor gene hCDC4 is frequently mutated in human T-cell acute lymphoblastic leukemia with functional consequences for Notch signaling. Cancer Res. (2007) 67:5611–6. doi: 10.1158/0008-5472.CAN-06-4381 17575125

[54] O'Neil J Grim J Strack P Rao S Tibbitts D Winter C. FBW7 mutations in leukemic cells mediate NOTCH pathway activation and resistance to gamma-secretase inhibitors. J Exp Med. (2007) 204:1813–24. doi: 10.1084/jem.20070876 PMC2118656 17646409

[55] Thompson BJ Buonamici S Sulis ML Palomero T Vilimas T Basso G. The SCFFBW7 ubiquitin ligase complex as a tumor suppressor in T cell leukemia. J Exp Med. (2007) 204:1825–35. doi: 10.1084/jem.20070872 PMC2118676 17646408

[56] Thompson BJ Jankovic V Gao J Buonamici S Vest A Lee JM. Control of hematopoietic stem cell quiescence by the E3 ubiquitin ligase Fbw7. J Exp Med. (2008) 205:1395–408. doi: 10.1084/jem.20080277 PMC2413036 18474632

[57] Reavie L Della Gatta G Crusio K Aranda-Orgilles B Buckley SM Thompson B. Regulation of hematopoietic stem cell differentiation by a single ubiquitin ligase-substrate complex. Nat Immunol. (2010) 11:207–15. doi: 10.1038/ni.1839 PMC2825759 20081848

[58] Fiore A Liang Y Lin YH Tung J Wang H Langlais D. Deubiquitinase MYSM1 in the hematopoietic system and beyond: A current review. Int J Mol Sci. (2020) 21. doi: 10.3390/ijms21083007 PMC7216186 32344625

[59] Nijnik A Clare S Hale C Raisen C McIntyre RE Yusa K. The critical role of histone H2A-deubiquitinase Mysm1 in hematopoiesis and lymphocyte differentiation. Blood. (2012) 119:1370–9. doi: 10.1182/blood-2011-05-352666 22184403

[60] Jiang XX Nguyen Q Chou Y Wang T Nandakumar V Yates P. Control of B cell development by the histone H2A deubiquitinase MYSM1. Immunity. (2011) 35:883–96. doi: 10.1016/j.immuni.2011.11.010 PMC4098839 22169041

[61] Reiley WW Zhang M Jin W Losiewicz M Donohue KB Norbury CC. Regulation of T cell development by the deubiquitinating enzyme CYLD. Nat Immunol. (2006) 7:411–7. doi: 10.1038/ni1315 16501569

[62] Panda S Nilsson JA Gekara NO. Deubiquitinase MYSM1 regulates innate immunity through inactivation of TRAF3 and TRAF6 complexes. Immunity. (2015) 43:647–59. doi: 10.1016/j.immuni.2015.09.010 26474655

[63] Panda S Gekara NO. The deubiquitinase MYSM1 dampens NOD2-mediated inflammation and tissue damage by inactivating the RIP2 complex. Nat Commun. (2018) 9:4654. doi: 10.1038/s41467-018-07016-0 30405132 PMC6220254

[64] Gatzka M Tasdogan A Hainzl A Allies G Maity P Wilms C. Interplay of H2A deubiquitinase 2A-DUB/Mysm1 and the p19(ARF)/p53 axis in hematopoiesis, early T-cell development and tissue differentiation. Cell Death Differ. (2015) 22:1451–62. doi: 10.1038/cdd.2014.231 PMC4532772 25613381

[65] Liu C Lan Y Liu B Zhang H Hu H. T cell development: old tales retold by single-cell RNA sequencing. Trends Immunol. (2021) 42:165–75. doi: 10.1016/j.it.2020.12.004 33446417

[66] Jones JM Simkus C. The roles of the RAG1 and RAG2 "non-core" regions in V(D)J recombination and lymphocyte development. Arch Immunol Ther Exp (Warsz). (2009) 57:105–16. doi: 10.1007/s00005-009-0011-3 19333736

[67] Ktorza S Blanc C Laurent C Sarun S Verpilleux MP Debré P. Complete TCR-delta rearrangements and partial (D-J) recombination of the TCR-beta locus in CD34+7+ precursors from human cord blood. J Immunol (Baltimore Md. 1950). (1996) 156:4120–7. doi: 10.4049/jimmunol.156.11.4120 8666778

[68] Yassai M Gorski J. Thymocyte maturation: selection for in-frame TCR alpha-chain rearrangement is followed by selection for shorter TCR beta-chain complementarity-determining region 3. J Immunol (Baltimore Md. 1950). (2000) 165:3706–12. doi: 10.4049/jimmunol.165.7.3706 11034375

[69] Michie AM Zuniga-Pflucker JC. Regulation of thymocyte differentiation: pre-TCR signals and beta-selection. Semin Immunol. (2002) 14:311–23. doi: 10.1016/s1044-5323(02)00064-7 12220932

[70] Voll RE Jimi E Phillips RJ Barber DF Rincon M Hayday AC. NF-kappa B activation by the pre-T cell receptor serves as a selective survival signal in T lymphocyte development. Immunity. (2000) 13:677–89. doi: 10.1016/s1074-7613(00)00067-4 11114380

[71] Aifantis I Gounari F Scorrano L Borowski C von Boehmer H. Constitutive pre-TCR signaling promotes differentiation through Ca2+ mobilization and activation of NF-kappaB and NFAT. Nat Immunol. (2001) 2:403–9. doi: 10.1038/87704 11323693

[72] Yamasaki S Ishikawa E Sakuma M Ogata K Sakata-Sogawa K Hiroshima M. Mechanistic basis of pre-T cell receptor-mediated autonomous signaling critical for thymocyte development. Nat Immunol. (2006) 7:67–75. doi: 10.1038/ni1290 16327787

[73] Aifantis I Buer J von Boehmer H Azogui O. Essential role of the pre-T cell receptor in allelic exclusion of the T cell receptor beta locus. Immunity. (1997) 7:601–7. doi: 10.1016/s1074-7613(00)80381-7 9390684

[74] Falk I Biro J Kohler H Eichmann K. Proliferation kinetics associated with T cell receptor-beta chain selection of fetal murine thymocytes. J Exp Med. (1996) 184:2327–39. doi: 10.1084/jem.184.6.2327 PMC2196377 8976187

[75] Yang-Iott KS Carpenter AC Rowh MA Steinel N Brady BL Hochedlinger K. TCR beta feedback signals inhibit the coupling of recombinationally accessible V beta 14 segments with DJ beta complexes. J Immunol (Baltimore Md. 1950). (2010) 184:1369–78. doi: 10.4049/jimmunol.0900723 PMC2873682 20042591

[76] von Boehmer H. Unique features of the pre-T-cell receptor alpha-chain: not just a surrogate. Nat Rev Immunol. (2005) 5:571–7. doi: 10.1038/nri1636 15999096

[77] Fehling HJ von Boehmer H. Early alpha beta T cell development in the thymus of normal and genetically altered mice. Curr Opin Immunol. (1997) 9:263–75. doi: 10.1016/S0952-7915(97)80146-X 9099797

[78] Trigueros C Ramiro AR Carrasco YR de Yebenes VG Albar JP Toribio ML. Identification of a late stage of small noncycling pTalpha- pre-T cells as immediate precursors of T cell receptor alpha/beta+ thymocytes. J Exp Med. (1998) 188:1401–12. doi: 10.1084/jem.188.8.1401 PMC2213418 9782117

[79] Carrasco YR Navarro MN Toribio ML. A role for the cytoplasmic tail of the pre-T cell receptor (TCR) alpha chain in promoting constitutive internalization and degradation of the pre-TCR. J Biol Chem. (2003) 278:14507–13. doi: 10.1074/jbc.M204944200 12473666

[80] Blom B Heemskerk MH Verschuren MC van Dongen JJ Stegmann AP Bakker AQ. Disruption of alpha beta but not of gamma delta T cell development by overexpression of the helix-loop-helix protein Id3 in committed T cell progenitors. EMBO J. (1999) 18:2793–802. doi: 10.1093/emboj/18.10.2793 PMC1171360 10329625

[81] Taghon T Van de Walle I De Smet G De Smedt M Leclercq G Vandekerckhove B. Notch signaling is required for proliferation but not for differentiation at a well-defined beta-selection checkpoint during human T-cell development. Blood. (2009) 113:3254–63. doi: 10.1182/blood-2008-07-168906 18948571

[82] Carrasco YR Navarro MN de Yebenes VG Ramiro AR Toribio ML. Regulation of surface expression of the human pre-T cell receptor complex. Semin Immunol. (2002) 14:325–34. doi: 10.1016/s1044-5323(02)00065-9 12220933

[83] Levelt CN Wang B Ehrfeld A Terhorst C Eichmann K. Regulation of T cell receptor (TCR)-beta locus allelic exclusion and initiation of TCR-alpha locus rearrangement in immature thymocytes by signaling through the CD3 complex. Eur J Immunol. (1995) 25:1257–61. doi: 10.1002/eji.1830250519 7774628

[84] Saito T Watanabe N. Positive and negative thymocyte selection. Crit Rev Immunol. (1998) 18:359–70. doi: 10.1615/critrevimmunol.v18.i4.40 9704194

[85] Klein L Kyewski B Allen PM Hogquist KA. Positive and negative selection of the T cell repertoire: what thymocytes see (and don't see). Nat Rev Immunol. (2014) 14:377–91. doi: 10.1038/nri3667 PMC4757912 24830344

[86] Yamashita I Nagata T Tada T Nakayama T. CD69 cell surface expression identifies developing thymocytes which audition for T cell antigen receptor-mediated positive selection. Int Immunol. (1993) 5:1139–50. doi: 10.1093/intimm/5.9.1139 7902130

[87] Ueno T Saito F Gray DH Kuse S Hieshima K Nakano H. CCR7 signals are essential for cortex-medulla migration of developing thymocytes. J Exp Med. (2004) 200:493–505. doi: 10.1084/jem.20040643 15302902 PMC2211934

[88] Kurobe H Liu C Ueno T Saito F Ohigashi I Seach N. CCR7-dependent cortex-to-medulla migration of positively selected thymocytes is essential for establishing central tolerance. Immunity. (2006) 24:165–77. doi: 10.1016/j.immuni.2005.12.011 16473829

[89] Nitta T Nitta S Lei Y Lipp M Takahama Y. CCR7-mediated migration of developing thymocytes to the medulla is essential for negative selection to tissue-restricted antigens. Proc Natl Acad Sci U.S.A. (2009) 106:17129–33. doi: 10.1073/pnas.0906956106 PMC2761327 19805112

[90] Brandle D Muller S Muller C Hengartner H Pircher H. Regulation of RAG-1 and CD69 expression in the thymus during positive and negative selection. Eur J Immunol. (1994) 24:145–51. doi: 10.1002/eji.1830240122 8020549

[91] Kyewski B Derbinski J. Self-representation in the thymus: an extended view. Nat Rev Immunol. (2004) 4:688–98. doi: 10.1038/nri1436 15343368

[92] Naramura M Jang IK Kole H Huang F Haines D Gu H. c-Cbl and Cbl-b regulate T cell responsiveness by promoting ligand-induced TCR down-modulation. Nat Immunol. (2002) 3:1192–9. doi: 10.1038/ni855 12415267

[93] Naramura M Kole HK Hu RJ Gu H. Altered thymic positive selection and intracellular signals in Cbl-deficient mice. Proc Natl Acad Sci U.S.A. (1998) 95:15547–52. doi: 10.1073/pnas.95.26.15547 PMC28080 9861006

[94] Wang HY Altman Y Fang D Elly C Dai Y Shao Y. Cbl promotes ubiquitination of the T cell receptor zeta through an adaptor function of Zap-70. J Biol Chem. (2001) 276:26004–11. doi: 10.1074/jbc.M010738200 11353765

[95] Chan S Correia-Neves M Benoist C Mathis D. CD4/CD8 lineage commitment: matching fate with competence. Immunol Rev. (1998) 165:195–207. doi: 10.1111/j.1600-065X.1998.tb01240.x 9850862

[96] Huang F Kitaura Y Jang I Naramura M Kole HH Liu L. Establishment of the major compatibility complex-dependent development of CD4+ and CD8+ T cells by the Cbl family proteins. Immunity. (2006) 25:571–81. doi: 10.1016/j.immuni.2006.08.021 17045823

[97] Groettrup M Ungewiss K Azogui O Palacios R Owen MJ Hayday AC. A novel disulfide-linked heterodimer on pre-T cells consists of the T cell receptor beta chain and a 33 kd glycoprotein. Cell. (1993) 75:283–94. doi: 10.1016/0092-8674(93)80070-U 8402912

[98] Berger MA Dave V Rhodes MR Bosma GC Bosma MJ Kappes DJ. Subunit composition of pre-T cell receptor complexes expressed by primary thymocytes: CD3 delta is physically associated but not functionally required. J Exp Med. (1997) 186:1461–7. doi: 10.1084/jem.186.9.1461 PMC2199111 9348303

[99] Panigada M Porcellini S Barbier E Hoeflinger S Cazenave PA Gu H. Constitutive endocytosis and degradation of the pre-T cell receptor. J Exp Med. (2002) 195:1585–97. doi: 10.1084/jem.20020047 PMC2193560 12070286

[100] Huang F Gu H. Negative regulation of lymphocyte development and function by the Cbl family of proteins. Immunol Rev. (2008) 224:229–38. doi: 10.1111/j.1600-065X.2008.00655.x 18759930

[101] Zhang J Stirling B Temmerman ST Ma CA Fuss IJ Derry JM. Impaired regulation of NF-kappaB and increased susceptibility to colitis-associated tumorigenesis in CYLD-deficient mice. J Clin Invest. (2006) 116:3042–9. doi: 10.1172/JCI28746 PMC1616194 17053834

[102] Tsagaratou A Trompouki E Grammenoudi S Kontoyiannis DL Mosialos G. Thymocyte-specific truncation of the deubiquitinating domain of CYLD impairs positive selection in a NF-kappaB essential modulator-dependent manner. J Immunol. (2010) 185:2032–43. doi: 10.4049/jimmunol.0903919 20644164

[103] Shim JH Xiao C Hayden MS Lee KY Trombetta ES Pypaert M. CHMP5 is essential for late endosome function and down-regulation of receptor signaling during mouse embryogenesis. J Cell Biol. (2006) 172:1045–56. doi: 10.1083/jcb.200509041 PMC2063762 16567502

[104] Rusten TE Vaccari T Stenmark H. Shaping development with ESCRTs. Nat Cell Biol. (2011) 14:38–45. doi: 10.1038/ncb2381 22193162

[105] Adoro S Park KH Bettigole SE Lis R Shin HR Seo H. Post-translational control of T cell development by the ESCRT protein CHMP5. Nat Immunol. (2017) 18:780–90. doi: 10.1038/ni.3764 28553951

[106] Linette GP Grusby MJ Hedrick SM Hansen TH Glimcher LH Korsmeyer SJ. Bcl-2 is upregulated at the CD4+ CD8+ stage during positive selection and promotes thymocyte differentiation at several control points. Immunity. (1994) 1:197–205. doi: 10.1016/1074-7613(94)90098-1 7889408

[107] Strasser A Harris AW von Boehmer H Cory S. Positive and negative selection of T cells in T-cell receptor transgenic mice expressing a bcl-2 transgene. Proc Natl Acad Sci U.S.A. (1994) 91:1376–80. doi: 10.1073/pnas.91.4.1376 PMC43161 8108419

[108] Starr TK Jameson SC Hogquist KA. Positive and negative selection of T cells. Annu Rev Immunol. (2003) 21:139–76. doi: 10.1146/annurev.immunol.21.120601.141107 12414722

[109] Azad N Iyer A Vallyathan V Wang L Castranova V Stehlik C. Role of oxidative/nitrosative stress-mediated Bcl-2 regulation in apoptosis and Malignant transformation. Ann N Y Acad Sci. (2010) 1203:1–6. doi: 10.1111/j.1749-6632.2010.05608.x 20716276

[110] Liu H Jain R Guan J Vuong V Ishido S La Gruta NL. Ubiquitin ligase MARCH 8 cooperates with CD83 to control surface MHC II expression in thymic epithelium and CD4 T cell selection. J Exp Med. (2016) 213:1695–703. doi: 10.1084/jem.20160312 PMC4995085 27503069

[111] von Rohrscheidt J Petrozziello E Nedjic J Federle C Krzyzak L Ploegh HL. Thymic CD4 T cell selection requires attenuation of March8-mediated MHCII turnover in cortical epithelial cells through CD83. J Exp Med. (2016) 213:1685–94. doi: 10.1084/jem.20160316 PMC4995086 27503071

[112] Prechtel AT Steinkasserer A. CD83: an update on functions and prospects of the maturation marker of dendritic cells. Arch Dermatol Res. (2007) 299:59–69. doi: 10.1007/s00403-007-0743-z 17334966

[113] Breloer M Fleischer B. CD83 regulates lymphocyte maturation, activation and homeostasis. Trends Immunol. (2008) 29:186–94. doi: 10.1016/j.it.2008.01.009 18329338

[114] Fujimoto Y Tu L Miller AS Bock C Fujimoto M Doyle C. CD83 expression influences CD4+ T cell development in the thymus. Cell. (2002) 108:755–67. doi: 10.1016/s0092-8674(02)00673-6 11955430

[115] Tze LE Horikawa K Domaschenz H Howard DR Roots CM Rigby RJ. CD83 increases MHC II and CD86 on dendritic cells by opposing IL-10-driven MARCH1-mediated ubiquitination and degradation. J Exp Med. (2011) 208:149–65. doi: 10.1084/jem.20092203 PMC3023131 21220452

[116] Emmerich CH Schmukle AC Walczak H. The emerging role of linear ubiquitination in cell signaling. Sci Signal. (2011) 4:re5. doi: 10.1126/scisignal.2002187 22375051

[117] Peltzer N Rieser E Taraborrelli L Draber P Darding M Pernaute B. HOIP deficiency causes embryonic lethality by aberrant TNFR1-mediated endothelial cell death. Cell Rep. (2014) 9:153–65. doi: 10.1016/j.celrep.2014.08.066 25284787

[118] Gerlach B Cordier SM Schmukle AC Emmerich CH Rieser E Haas TL. Linear ubiquitination prevents inflammation and regulates immune signalling. Nature. (2011) 471:591–6. doi: 10.1038/nature09816 21455173

[119] Boisson B Laplantine E Dobbs K Cobat A Tarantino N Hazen M. Human HOIP and LUBAC deficiency underlies autoinflammation, immunodeficiency, amylopectinosis, and lymphangiectasia. J Exp Med. (2015) 212:939–51. doi: 10.1084/jem.20141130 PMC4451137 26008899

[120] Teh CE Lalaoui N Jain R Policheni AN Heinlein M Alvarez-Diaz S. Linear ubiquitin chain assembly complex coordinates late thymic T-cell differentiation and regulatory T-cell homeostasis. Nat Commun. (2016) 7:13353. doi: 10.1038/ncomms13353 27857075 PMC5120208

[121] Owen DL Mahmud SA Sjaastad LE Williams JB Spanier JA Simeonov DR. Thymic regulatory T cells arise via two distinct developmental programs. Nat Immunol. (2019) 20:195–205. doi: 10.1038/s41590-018-0289-6 30643267 PMC6650268

[122] Lio CW Hsieh CS. A two-step process for thymic regulatory T cell development. Immunity. (2008) 28:100–11. doi: 10.1016/j.immuni.2007.11.021 PMC2248212 18199417

[123] Tai X Erman B Alag A Mu J Kimura M Katz G. Foxp3 transcription factor is proapoptotic and lethal to developing regulatory T cells unless counterbalanced by cytokine survival signals. Immunity. (2013) 38:1116–28. doi: 10.1016/j.immuni.2013.02.022 PMC3700677 23746651

[124] Long M Park SG Strickland I Hayden MS Ghosh S. Nuclear factor-kappaB modulates regulatory T cell development by directly regulating expression of Foxp3 transcription factor. Immunity. (2009) 31:921–31. doi: 10.1016/j.immuni.2009.09.022 20064449

[125] Shortman K Egerton M Spangrude GJ Scollay R. The generation and fate of thymocytes. Semin Immunol. (1990) 2:3–12. 2129900

[126] Penit C Lucas B Vasseur F. Cell expansion and growth arrest phases during the transition from precursor (CD4-8-) to immature (CD4+8+) thymocytes in normal and genetically modified mice. J Immunol. (1995) 154:5103–13. doi: 10.4049/jimmunol.154.10.5103 7730616

[127] Rodewald HR Ogawa M Haller C Waskow C DiSanto JP. Pro-thymocyte expansion by c-kit and the common cytokine receptor gamma chain is essential for repertoire formation. Immunity. (1997) 6:265–72. doi: 10.1016/s1074-7613(00)80329-5 9075927

[128] Mazzucchelli R Durum SK. Interleukin-7 receptor expression: intelligent design. Nat Rev Immunol. (2007) 7:144–54. doi: 10.1038/nri2023 17259970

[129] Boudil A Matei IR Shih H-Y Bogdanoski G Yuan JS Chang SG. IL-7 coordinates proliferation, differentiation and Tcra recombination during thymocyte β-selection. Nat Immunol. (2015) 16:397–405. doi: 10.1038/ni.3122 25729925 PMC4368453

[130] Kreslavsky T Gleimer M Miyazaki M Choi Y Gagnon E Murre C. beta-Selection-induced proliferation is required for alphabeta T cell differentiation. Immunity. (2012) 37:840–53. doi: 10.1016/j.immuni.2012.08.020 PMC3709258 23159226

[131] Hoffman ES Passoni L Crompton T Leu TM Schatz DG Koff A. Productive T-cell receptor beta-chain gene rearrangement: coincident regulation of cell cycle and clonality during development in *vivo* . Genes Dev. (1996) 10:948–62. doi: 10.1101/gad.10.8.948 8608942

[132] Aifantis I Mandal M Sawai K Ferrando A Vilimas T. Regulation of T-cell progenitor survival and cell-cycle entry by the pre-T-cell receptor. Immunol Rev. (2006) 209:159–69. doi: 10.1111/j.0105-2896.2006.00343.x 16448541

[133] Rowell EA Wells AD. The role of cyclin-dependent kinases in T-cell development, proliferation, and function. Crit Rev Immunol. (2006) 26:189–212. doi: 10.1615/critrevimmunol.v26.i3.10 16928186

[134] Nakayama K Ishida N Shirane M Inomata A Inoue T Shishido N. Mice lacking p27(Kip1) display increased body size, multiple organ hyperplasia, retinal dysplasia, and pituitary tumors. Cell. (1996) 85:707–20. doi: 10.1016/s0092-8674(00)81237-4 8646779

[135] Kiyokawa H Kineman RD Manova-Todorova KO Soares VC Hoffman ES Ono M. Enhanced growth of mice lacking the cyclin-dependent kinase inhibitor function of p27(Kip1). Cell. (1996) 85:721–32. doi: 10.1016/s0092-8674(00)81238-6 8646780

[136] Fero ML Rivkin M Tasch M Porter P Carow CE Firpo E. A syndrome of multiorgan hyperplasia with features of gigantism, tumorigenesis, and female sterility in p27(Kip1)-deficient mice. Cell. (1996) 85:733–44. doi: 10.1016/s0092-8674(00)81239-8 8646781

[137] Tsukiyama T Ishida N Shirane M Minamishima YA Hatakeyama S Kitagawa M. Down-regulation of p27Kip1 expression is required for development and function of T cells. J Immunol. (2001) 166:304–12. doi: 10.4049/jimmunol.166.1.304 11123306

[138] Xu S Abbasian M Patel P Jensen-Pergakes K Lombardo CR Cathers BE. Substrate recognition and ubiquitination of SCFSkp2/Cks1 ubiquitin-protein isopeptide ligase. J Biol Chem. (2007) 282:15462–70. doi: 10.1074/jbc.M610758200 17409098

[139] Hao B Zheng N Schulman BA Wu G Miller JJ Pagano M. Structural basis of the Cks1-dependent recognition of p27(Kip1) by the SCF(Skp2) ubiquitin ligase. Mol Cell. (2005) 20:9–19. doi: 10.1016/j.molcel.2005.09.003 16209941

[140] Zhao B Yoganathan K Li L Lee JY Zuniga-Pflucker JC Love PE. Notch and the pre-TCR coordinate thymocyte proliferation by induction of the SCF subunits Fbxl1 and Fbxl12. Nat Immunol. (2019) 20:1381–92. doi: 10.1038/s41590-019-0469-z PMC6754294 31451788

[141] Sarmento LM Huang H Limon A Gordon W Fernandes J Tavares MJ. Notch1 modulates timing of G1-S progression by inducing SKP2 transcription and p27 Kip1 degradation. J Exp Med. (2005) 202:157–68. doi: 10.1084/jem.20050559 PMC2212905 15998794

[142] Kossatz U Dietrich N Zender L Buer J Manns MP Malek NP. Skp2-dependent degradation of p27kip1 is essential for cell cycle progression. Genes Dev. (2004) 18:2602–7. doi: 10.1101/gad.321004 PMC525540 15520280

[143] Tsvetkov LM Yeh KH Lee SJ Sun H Zhang H. p27(Kip1) ubiquitination and degradation is regulated by the SCF(Skp2) complex through phosphorylated Thr187 in p27. Curr Biol. (1999) 9:661–4. doi: 10.1016/s0960-9822(99)80290-5 10375532

[144] Nakayama K Nagahama H Minamishima YA Miyake S Ishida N Hatakeyama S. Skp2-mediated degradation of p27 regulates progression into mitosis. Dev Cell. (2004) 6:661–72. doi: 10.1016/s1534-5807(04)00131-5 15130491

[145] Nishiyama M Nita A Yumimoto K Nakayama KI. FBXL12-mediated degradation of ALDH3 is essential for trophoblast differentiation during placental development. Stem Cells. (2015) 33:3327–40. doi: 10.1002/stem.2088 26124079

[146] Dufner A Kisser A Niendorf S Basters A Reissig S Schonle A. The ubiquitin-specific protease USP8 is critical for the development and homeostasis of T cells. Nat Immunol. (2015) 16:950–60. doi: 10.1038/ni.3230 26214742

[147] Kerdiles YM Beisner DR Tinoco R Dejean AS Castrillon DH DePinho RA. Foxo1 links homing and survival of naive T cells by regulating L-selectin, CCR7 and interleukin 7 receptor. Nat Immunol. (2009) 10:176–84. doi: 10.1038/ni.1689 PMC2856471 19136962

[148] Ouyang W Beckett O Flavell RA Li MO. An essential role of the Forkhead-box transcription factor Foxo1 in control of T cell homeostasis and tolerance. Immunity. (2009) 30:358–71. doi: 10.1016/j.immuni.2009.02.003 PMC2692529 19285438

[149] Tzivion G Dobson M Ramakrishnan G. FoxO transcription factors; Regulation by AKT and 14-3-3 proteins. Biochim Biophys Acta. (2011) 1813:1938–45. doi: 10.1016/j.bbamcr.2011.06.002 21708191

[150] Gossage L Eisen T Maher ER. VHL, the story of a tumour suppressor gene. Nat Rev Cancer. (2015) 15:55–64. doi: 10.1038/nrc3844 25533676

[151] Hon WC Wilson MI Harlos K Claridge TD Schofield CJ Pugh CW. Structural basis for the recognition of hydroxyproline in HIF-1 alpha by pVHL. Nature. (2002) 417:975–8. doi: 10.1038/nature00767 12050673

[152] Gnarra JR Tory K Weng Y Schmidt L Wei MH Li H. Mutations of the VHL tumour suppressor gene in renal carcinoma. Nat Genet. (1994) 7:85–90. doi: 10.1038/ng0594-85 7915601

[153] Biju MP Neumann AK Bensinger SJ Johnson RS Turka LA Haase VH. Vhlh gene deletion induces Hif-1-mediated cell death in thymocytes. Mol Cell Biol. (2004) 24:9038–47. doi: 10.1128/mcb.24.20.9038-9047.2004 PMC517905 15456877

[154] Jaakkola P Mole DR Tian YM Wilson MI Gielbert J Gaskell SJ. Targeting of HIF-alpha to the von Hippel-Lindau ubiquitylation complex by O2-regulated prolyl hydroxylation. Science. (2001) 292:468–72. doi: 10.1126/science.1059796 11292861

[155] Savage PA Klawon DEJ Miller CH. Regulatory T cell development. Annu Rev Immunol. (2020) 38:421–53. doi: 10.1146/annurev-immunol-100219-020937 31990619

[156] Caramalho I Nunes-Cabaco H Foxall RB Sousa AE. Regulatory T-cell development in the human thymus. Front Immunol. (2015) 6:395. doi: 10.3389/fimmu.2015.00395 26284077 PMC4522873

[157] Aschenbrenner K D'Cruz LM Vollmann EH Hinterberger M Emmerich J Swee LK. Selection of Foxp3+ regulatory T cells specific for self antigen expressed and presented by Aire+ medullary thymic epithelial cells. Nat Immunol. (2007) 8:351–8. doi: 10.1038/ni1444 17322887

[158] Fontenot JD Gavin MA Rudensky AY. Foxp3 programs the development and function of CD4+CD25+ regulatory T cells. Nat Immunol. (2003) 4:330–6. doi: 10.1038/ni904 12612578

[159] Fontenot JD Rasmussen JP Gavin MA Rudensky AY. A function for interleukin 2 in Foxp3-expressing regulatory T cells. Nat Immunol. (2005) 6:1142–51. doi: 10.1038/ni1263 16227984

[160] Tai X Cowan M Feigenbaum L Singer A. CD28 costimulation of developing thymocytes induces Foxp3 expression and regulatory T cell differentiation independently of interleukin 2. Nat Immunol. (2005) 6:152–62. doi: 10.1038/ni1160 15640801

[161] Richards DM Delacher M Goldfarb Y Kagebein D Hofer AC Abramson J. Treg cell differentiation: from thymus to peripheral tissue. Prog Mol Biol Transl Sci. (2015) 136:175–205. doi: 10.1016/bs.pmbts.2015.07.014 26615097

[162] Lee HM Bautista JL Hsieh CS. Thymic and peripheral differentiation of regulatory T cells. Adv Immunol. (2011) 112:25–71. doi: 10.1016/B978-0-12-387827-4.00002-4 22118406

[163] Itoh M Takahashi T Sakaguchi N Kuniyasu Y Shimizu J Otsuka F. Thymus and autoimmunity: production of CD25+CD4+ naturally anergic and suppressive T cells as a key function of the thymus in maintaining immunologic self-tolerance. J Immunol (Baltimore Md. 1950). (1999) 162:5317–26. doi: 10.4049/jimmunol.162.9.5317 10228007

[164] Burchill MA Yang J Vang KB Moon JJ Chu HH Lio CW. Linked T cell receptor and cytokine signaling govern the development of the regulatory T cell repertoire. Immunity. (2008) 28:112–21. doi: 10.1016/j.immuni.2007.11.022 PMC2430111 18199418

[165] Palmer E Naeher D. Affinity threshold for thymic selection through a T-cell receptor–co-receptor zipper. Nat Rev Immunol. (2009) 9:207–13. doi: 10.1038/nri2469 19151748

[166] Pacholczyk R Ignatowicz H Kraj P Ignatowicz L. Origin and T cell receptor diversity of Foxp3+CD4+CD25+ T cells. Immunity. (2006) 25:249–59. doi: 10.1016/j.immuni.2006.05.016 16879995

[167] Picca CC Larkin J 3rd Boesteanu A Lerman MA Rankin AL Caton AJ. Role of TCR specificity in CD4+ CD25+ regulatory T-cell selection. Immunol Rev. (2006) 212:74–85. doi: 10.1111/j.0105-2896.2006.00416.x 16903907

[168] Cabarrocas J Cassan C Magnusson F Piaggio E Mars L Derbinski J. Foxp3+ CD25+ regulatory T cells specific for a neo-self-antigen develop at the double-positive thymic stage. Proc Natl Acad Sci U.S.A. (2006) 103:8453–8. doi: 10.1073/pnas.0603086103 PMC1482513 16709665

[169] Jordan MS Boesteanu A Reed AJ Petrone AL Holenbeck AE Lerman MA. Thymic selection of CD4+CD25+ regulatory T cells induced by an agonist self-peptide. Nat Immunol. (2001) 2:301–6. doi: 10.1038/86302 11276200

[170] Caton AJ Kropf E Simons DM Aitken M Weissler KA Jordan MS. Strength of TCR signal from self-peptide modulates autoreactive thymocyte deletion and Foxp3(+) Treg-cell formation. Eur J Immunol. (2014) 44:785–93. doi: 10.1002/eji.201343767 PMC3959276 24307208

[171] Larkin J 3rd Rankin AL Picca CC Riley MP Jenks SA Sant AJ. CD4+CD25+ regulatory T cell repertoire formation shaped by differential presentation of peptides from a self-antigen. J Immunol (Baltimore Md. 1950). (2008) 180:2149–57. doi: 10.4049/jimmunol.180.4.2149 18250421

[172] Weissler KA Caton AJ. The role of T-cell receptor recognition of peptide:MHC complexes in the formation and activity of Foxp3(+) regulatory T cells. Immunol Rev. (2014) 259:11–22. doi: 10.1111/imr.12177 24712456 PMC4034456

[173] Guo F Iclozan C Suh WK Anasetti C Yu XZ. CD28 controls differentiation of regulatory T cells from naive CD4 T cells. J Immunol (Baltimore Md. 1950). (2008) 181:2285–91. doi: 10.4049/jimmunol.181.4.2285 PMC2688779 18684917

[174] Legrand N Cupedo T van Lent AU Ebeli MJ Weijer K Hanke T. Transient accumulation of human mature thymocytes and regulatory T cells with CD28 superagonist in "human immune system" Rag2(-/-)gammac(-/-) mice. Blood. (2006) 108:238–45. doi: 10.1182/blood-2006-01-0190 16514056

[175] Poliani PL Fontana E Roifman CM Notarangelo LD. zeta Chain-associated protein of 70 kDa (ZAP70) deficiency in human subjects is associated with abnormalities of thymic stromal cells: Implications for T-cell tolerance. J Allergy Clin Immunol. (2013) 131:597–600.e591-592. doi: 10.1016/j.jaci.2012.11.002 23245794

[176] Bachmaier K Krawczyk C Kozieradzki I Kong YY Sasaki T Oliveira-dos-Santos A. Negative regulation of lymphocyte activation and autoimmunity by the molecular adaptor Cbl-b. Nature. (2000) 403:211–6. doi: 10.1038/35003228 10646608

[177] Chiang YJ Kole HK Brown K Naramura M Fukuhara S Hu RJ. Cbl-b regulates the CD28 dependence of T-cell activation. Nature. (2000) 403:216–20. doi: 10.1038/35003235 10646609

[178] Zhao Y Guo H Qiao G Zucker M Langdon WY Zhang J. E3 ubiquitin ligase cbl-b regulates thymic-derived CD4+CD25+ Regulatory T cell development by targeting foxp3 for ubiquitination. J Immunol. (2015) 194:1639–45. doi: 10.4049/jimmunol.1402434 PMC4324371 25560411

[179] Harada Y Harada Y Elly C Ying G Paik JH DePinho RA. Transcription factors Foxo3a and Foxo1 couple the E3 ligase Cbl-b to the induction of Foxp3 expression in induced regulatory T cells. J Exp Med. (2010) 207:1381–91. doi: 10.1084/jem.20100004 PMC2901074 20439537

[180] Wohlfert EA Gorelik L Mittler R Flavell RA Clark RB. Cutting edge: deficiency in the E3 ubiquitin ligase Cbl-b results in a multifunctional defect in T cell TGF-beta sensitivity. Vitro vivo. J Immunol. (2006) 176:1316–20. doi: 10.4049/jimmunol.176.3.1316 16424156

[181] Qiao G Zhao Y Li Z Tang PQ Langdon WY Yang T. T cell activation threshold regulated by E3 ubiquitin ligase Cbl-b determines fate of inducible regulatory T cells. J Immunol. (2013) 191:632–9. doi: 10.4049/jimmunol.1202068 PMC3702637 23749633

[182] Tai X Indart A Rojano M Guo J Apenes N Kadakia T. How autoreactive thymocytes differentiate into regulatory versus effector CD4(+) T cells after avoiding clonal deletion. Nat Immunol. (2023) 24:637–51. doi: 10.1038/s41590-023-01469-2 PMC10063450 36959291

[183] Qiao G Li Z Molinero L Alegre ML Ying H Sun Z. T-cell receptor-induced NF-kappaB activation is negatively regulated by E3 ubiquitin ligase Cbl-b. Mol Cell Biol. (2008) 28:2470–80. doi: 10.1128/mcb.01505-07 PMC2268433 18227156

[184] Hsieh CS Lee HM Lio CW. Selection of regulatory T cells in the thymus. Nat Rev Immunol. (2012) 12:157–67. doi: 10.1038/nri3155 22322317

[185] Laufer TM DeKoning J Markowitz JS Lo D Glimcher LH. Unopposed positive selection and autoreactivity in mice expressing class II MHC only on thymic cortex. Nature. (1996) 383:81–5. doi: 10.1038/383081a0 8779719

[186] Baravalle G Park H McSweeney M Ohmura-Hoshino M Matsuki Y Ishido S. Ubiquitination of CD86 is a key mechanism in regulating antigen presentation by dendritic cells. J Immunol. (2011) 187:2966–73. doi: 10.4049/jimmunol.1101643 PMC4496154 21849678

[187] Proietto AI van Dommelen S Zhou P Rizzitelli A D'Amico A Steptoe RJ. Dendritic cells in the thymus contribute to T-regulatory cell induction. Proc Natl Acad Sci U.S.A. (2008) 105:19869–74. doi: 10.1073/pnas.0810268105 PMC2604962 19073916

[188] Matsuki Y Ohmura-Hoshino M Goto E Aoki M Mito-Yoshida M Uematsu M. Novel regulation of MHC class II function in B cells. EMBO J. (2007) 26:846–54. doi: 10.1038/sj.emboj.7601556 PMC1794403 17255932

[189] De Gassart A Camosseto V Thibodeau J Ceppi M Catalan N Pierre P. MHC class II stabilization at the surface of human dendritic cells is the result of maturation-dependent MARCH I down-regulation. Proc Natl Acad Sci U.S.A. (2008) 105:3491–6. doi: 10.1073/pnas.0708874105 PMC2265198 18305173

[190] Walseng E Furuta K Goldszmid RS Weih KA Sher A Roche PA. Dendritic cell activation prevents MHC class II ubiquitination and promotes MHC class II survival regardless of the activation stimulus. J Biol Chem. (2010) 285:41749–54. doi: 10.1074/jbc.M110.157586 PMC3009902 21047782

[191] Oh J Wu N Baravalle G Cohn B Ma J Lo B. MARCH1-mediated MHCII ubiquitination promotes dendritic cell selection of natural regulatory T cells. J Exp Med. (2013) 210:1069–77. doi: 10.1084/jem.20122695 PMC3674695 23712430

[192] Liu H Wilson KR Schriek P Macri C Blum AB Francis L. Ubiquitination of MHC class II is required for development of regulatory but not conventional CD4(+) T cells. J Immunol. (2020) 205:1207–16. doi: 10.4049/jimmunol.1901328 32747505

[193] Liu YC. The E3 ubiquitin ligase Itch in T cell activation, differentiation, and tolerance. Semin Immunol. (2007) 19:197–205. doi: 10.1016/j.smim.2007.02.003 17433711 PMC2680672

[194] Venuprasad K Huang H Harada Y Elly C Subramaniam M Spelsberg T. The E3 ubiquitin ligase Itch regulates expression of transcription factor Foxp3 and airway inflammation by enhancing the function of transcription factor TIEG1. Nat Immunol. (2008) 9:245–53. doi: 10.1038/ni1564 PMC2610020 18278048

[195] Beal AM Ramos-Hernandez N Riling CR Nowelsky EA Oliver PM. TGF-beta induces the expression of the adaptor Ndfip1 to silence IL-4 production during iTreg cell differentiation. Nat Immunol. (2011) 13:77–85. doi: 10.1038/ni.2154 22080920 PMC3542978

[196] Fang D Elly C Gao B Fang N Altman Y Joazeiro C. Dysregulation of T lymphocyte function in itchy mice: a role for Itch in TH2 differentiation. Nat Immunol. (2002) 3:281–7. doi: 10.1038/ni763 11828324

[197] Venuprasad K Elly C Gao M Salek-Ardakani S Harada Y Luo JL. Convergence of Itch-induced ubiquitination with MEKK1-JNK signaling in Th2 tolerance and airway inflammation. J Clin Invest. (2006) 116:1117–26. doi: 10.1172/JCI26858 PMC1409741 16557301

[198] Gao M Labuda T Xia Y Gallagher E Fang D Liu YC. Jun turnover is controlled through JNK-dependent phosphorylation of the E3 ligase Itch. Science. (2004) 306:271–5. doi: 10.1126/science.1099414 15358865

[199] Oliver PM Cao X Worthen GS Shi P Briones N MacLeod M. Ndfip1 protein promotes the function of itch ubiquitin ligase to prevent T cell activation and T helper 2 cell-mediated inflammation. Immunity. (2006) 25:929–40. doi: 10.1016/j.immuni.2006.10.012 PMC2955961 17137798

[200] Ramon HE Riling CR Bradfield J Yang B Hakonarson H Oliver PM. The ubiquitin ligase adaptor Ndfip1 regulates T cell-mediated gastrointestinal inflammation and inflammatory bowel disease susceptibility. Mucosal Immunol. (2011) 4:314–24. doi: 10.1038/mi.2010.69 PMC3905456 20962770

[201] Dardalhon V Awasthi A Kwon H Galileos G Gao W Sobel RA. IL-4 inhibits TGF-beta-induced Foxp3+ T cells and, together with TGF-beta, generates IL-9+ IL-10+ Foxp3(-) effector T cells. Nat Immunol. (2008) 9:1347–55. doi: 10.1038/ni.1677 PMC2999006 18997793

[202] Hacker H Tseng PH Karin M. Expanding TRAF function: TRAF3 as a tri-faced immune regulator. Nat Rev Immunol. (2011) 11:457–68. doi: 10.1038/nri2998 21660053

[203] Yi Z Lin WW Stunz LL Bishop GA. The adaptor TRAF3 restrains the lineage determination of thymic regulatory T cells by modulating signaling via the receptor for IL-2. Nat Immunol. (2014) 15:866–74. doi: 10.1038/ni.2944 PMC4139452 25029551

[204] Xie P Kraus ZJ Stunz LL Liu Y Bishop GA. TNF receptor-associated factor 3 is required for T cell-mediated immunity and TCR/CD28 signaling. J Immunol. (2011) 186:143–55. doi: 10.4049/jimmunol.1000290 PMC3044490 21084666

[205] Malek TR Castro I. Interleukin-2 receptor signaling: at the interface between tolerance and immunity. Immunity. (2010) 33:153–65. doi: 10.1016/j.immuni.2010.08.004 PMC2946796 20732639

[206] Johnston JA Bacon CM Riedy MC O'Shea JJ. Signaling by IL-2 and related cytokines: JAKs, STATs, and relationship to immunodeficiency. J Leukoc Biol. (1996) 60:441–52. doi: 10.1002/jlb.60.4.441 8864127

[207] Minami Y Taniguchi T. IL-2 signaling: recruitment and activation of multiple protein tyrosine kinases by the components of the IL-2 receptor. Curr Opin Cell Biol. (1995) 7:156–62. doi: 10.1016/0955-0674(95)80023-9 7612266

[208] Burchill MA Yang J Vogtenhuber C Blazar BR Farrar MA. IL-2 receptor beta-dependent STAT5 activation is required for the development of Foxp3+ regulatory T cells. J Immunol. (2007) 178:280–90. doi: 10.4049/jimmunol.178.1.280 17182565

[209] Yao Z Kanno Y Kerenyi M Stephens G Durant L Watford WT. Nonredundant roles for Stat5a/b in directly regulating Foxp3. Blood. (2007) 109:4368–75. doi: 10.1182/blood-2006-11-055756 PMC1885496 17227828

[210] Chang JH Hu H Jin J Puebla-Osorio N Xiao Y Gilbert BE. TRAF3 regulates the effector function of regulatory T cells and humoral immune responses. J Exp Med. (2014) 211:137–51. doi: 10.1084/jem.20131019 PMC3892978 24378539

[211] Ni X Kou W Gu J Wei P Wu X Peng H. TRAF6 directs FOXP3 localization and facilitates regulatory T-cell function through K63-linked ubiquitination. EMBO J. (2019) 38. doi: 10.15252/embj.201899766 PMC6484404 30886050

[212] Vereecke L Beyaert R van Loo G. The ubiquitin-editing enzyme A20 (TNFAIP3) is a central regulator of immunopathology. Trends Immunol. (2009) 30:383–91. doi: 10.1016/j.it.2009.05.007 19643665

[213] Coornaert B Baens M Heyninck K Bekaert T Haegman M Staal J. T cell antigen receptor stimulation induces MALT1 paracaspase-mediated cleavage of the NF-kappaB inhibitor A20. Nat Immunol. (2008) 9:263–71. doi: 10.1038/ni1561 18223652

[214] Duwel M Welteke V Oeckinghaus A Baens M Kloo B Ferch U. A20 negatively regulates T cell receptor signaling to NF-kappaB by cleaving Malt1 ubiquitin chains. J Immunol. (2009) 182:7718–28. doi: 10.4049/jimmunol.0803313 19494296

[215] Onizawa M Oshima S Schulze-Topphoff U Oses-Prieto JA Lu T Tavares R. The ubiquitin-modifying enzyme A20 restricts ubiquitination of the kinase RIPK3 and protects cells from necroptosis. Nat Immunol. (2015) 16:618–27. doi: 10.1038/ni.3172 PMC4439357 25939025

[216] Fischer JC Otten V Kober M Drees C Rosenbaum M Schmickl M. A20 restrains thymic regulatory T cell development. J Immunol. (2017) 199:2356–65. doi: 10.4049/jimmunol.1602102 PMC5617121 28842469

[217] Mahmud SA Manlove LS Schmitz HM Xing Y Wang Y Owen DL. Costimulation via the tumor-necrosis factor receptor superfamily couples TCR signal strength to the thymic differentiation of regulatory T cells. Nat Immunol. (2014) 15:473–81. doi: 10.1038/ni.2849 PMC4000541 24633226

[218] Zhao Y Thornton AM Kinney MC Ma CA Spinner JJ Fuss IJ. The deubiquitinase CYLD targets Smad7 protein to regulate transforming growth factor beta (TGF-beta) signaling and the development of regulatory T cells. J Biol Chem. (2011) 286:40520–30. doi: 10.1074/jbc.M111.292961 PMC3220473 21931165

[219] Hovelmeyer N Wunderlich FT Massoumi R Jakobsen CG Song J Worns MA. Regulation of B cell homeostasis and activation by the tumor suppressor gene CYLD. J Exp Med. (2007) 204:2615–27. doi: 10.1084/jem.20070318 PMC2118471 17923499

[220] Reissig S Hovelmeyer N Weigmann B Nikolaev A Kalt B Wunderlich TF. The tumor suppressor CYLD controls the function of murine regulatory T cells. J Immunol. (2012) 189:4770–6. doi: 10.4049/jimmunol.1201993 23066153

[221] Ladi E Yin X Chtanova T Robey EA. Thymic microenvironments for T cell differentiation and selection. Nat Immunol. (2006) 7:338–43. doi: 10.1038/ni1323 16550196

[222] Han J Zúñiga-Pflücker JC. A 2020 View of thymus stromal cells in T cell development. J Immunol (Baltimore Md. 1950). (2021) 206:249–56. doi: 10.4049/jimmunol.2000889 PMC7909612 33397738

[223] Cosway EJ Lucas B James KD Parnell SM Carvalho-Gaspar M White AJ. Redefining thymus medulla specialization for central tolerance. J Exp Med. (2017) 214:3183–95. doi: 10.1084/jem.20171000 PMC5679166 28830910

[224] Kadouri N Nevo S Goldfarb Y Abramson J. Thymic epithelial cell heterogeneity: TEC by TEC. Nat Rev Immunol. (2020) 20:239–53. doi: 10.1038/s41577-019-0238-0 31804611

[225] Anderson G Takahama Y. Thymic epithelial cells: working class heroes for T cell development and repertoire selection. Trends Immunol. (2012) 33:256–63. doi: 10.1016/j.it.2012.03.005 22591984

[226] Abramson J Anderson G. Thymic epithelial cells. Annu Rev Immunol. (2017) 35:85–118. doi: 10.1146/annurev-immunol-051116-052320 28226225

[227] Takahama Y Ohigashi I Baik S Anderson G. Generation of diversity in thymic epithelial cells. Nat Rev Immunol. (2017) 17:295–305. doi: 10.1038/nri.2017.12 28317923

[228] Wang HX Pan W Zheng L Zhong XP Tan L Liang Z. Thymic epithelial cells contribute to thymopoiesis and T cell development. Front Immunol. (2019) 10:3099. doi: 10.3389/fimmu.2019.03099 32082299 PMC7005006

[229] Derbinski J Schulte A Kyewski B Klein L. Promiscuous gene expression in medullary thymic epithelial cells mirrors the peripheral self. Nat Immunol. (2001) 2:1032–9. doi: 10.1038/ni723 11600886

[230] Danan-Gotthold M Guyon C Giraud M Levanon EY Abramson J. Extensive RNA editing and splicing increase immune self-representation diversity in medullary thymic epithelial cells. Genome Biol. (2016) 17:219. doi: 10.1186/s13059-016-1079-9 27776542 PMC5078920

[231] Mathis D Benoist C. Back to central tolerance. Immunity. (2004) 20:509–16. doi: 10.1016/s1074-7613(04)00111-6 15142520

[232] Marx A Yamada Y Simon-Keller K Schalke B Willcox N Strobel P. Thymus and autoimmunity. Semin Immunopathol. (2021) 43:45–64. doi: 10.1007/s00281-021-00842-3 33537838 PMC7925479

[233] Anderson MS Venanzi ES Klein L Chen Z Berzins SP Turley SJ. Projection of an immunological self shadow within the thymus by the aire protein. Science. (2002) 298:1395–401. doi: 10.1126/science.1075958 12376594

[234] Anderson MS Venanzi ES Chen Z Berzins SP Benoist C Mathis D. The cellular mechanism of Aire control of T cell tolerance. Immunity. (2005) 23:227–39. doi: 10.1016/j.immuni.2005.07.005 16111640

[235] Perry JSA Lio CJ Kau AL Nutsch K Yang Z Gordon JI. Distinct contributions of Aire and antigen-presenting-cell subsets to the generation of self-tolerance in the thymus. Immunity. (2014) 41:414–26. doi: 10.1016/j.immuni.2014.08.007 PMC4175925 25220213

[236] Oftedal BE Hellesen A Erichsen MM Bratland E Vardi A Perheentupa J. Dominant mutations in the autoimmune regulator AIRE are associated with common organ-specific autoimmune diseases. Immunity. (2015) 42:1185–96. doi: 10.1016/j.immuni.2015.04.021 26084028

[237] Nagamine K Peterson P Scott HS Kudoh J Minoshima S Heino M. Positional cloning of the APECED gene. Nat Genet. (1997) 17:393–8. doi: 10.1038/ng1297-393 9398839

[238] Zhu W Hu Z Liao X Chen X Huang W Zhong Y. A new mutation site in the AIRE gene causes autoimmune polyendocrine syndrome type 1. Immunogenetics. (2017) 69:643–51. doi: 10.1007/s00251-017-0995-5 28540407

[239] Kumar PG Laloraya M Wang CY Ruan QG Davoodi-Semiromi A Kao KJ. The autoimmune regulator (AIRE) is a DNA-binding protein. J Biol Chem. (2001) 276:41357–64. doi: 10.1074/jbc.M104898200 11533054

[240] Pascual J Martinez-Yamout M Dyson HJ Wright PE. Structure of the PHD zinc finger from human Williams-Beuren syndrome transcription factor. J Mol Biol. (2000) 304:723–9. doi: 10.1006/jmbi.2000.4308 11124022

[241] Uchida D Hatakeyama S Matsushima A Han H Ishido S Hotta H. AIRE functions as an E3 ubiquitin ligase. J Exp Med. (2004) 199:167–72. doi: 10.1084/jem.20031291 PMC2211764 14734522

[242] Bottomley MJ Stier G Pennacchini D Legube G Simon B Akhtar A. NMR structure of the first PHD finger of autoimmune regulator protein (AIRE1). Insights into autoimmune polyendocrinopathy-candidiasis-ectodermal dystrophy (APECED) disease. J Biol Chem. (2005) 280:11505–12. doi: 10.1074/jbc.M413959200 15649886

[243] Naito A Azuma S Tanaka S Miyazaki T Takaki S Takatsu K. Severe osteopetrosis, defective interleukin-1 signalling and lymph node organogenesis in TRAF6-deficient mice. Genes Cells. (1999) 4:353–62. doi: 10.1046/j.1365-2443.1999.00265.x 10421844

[244] Akiyama T Maeda S Yamane S Ogino K Kasai M Kajiura F. Dependence of self-tolerance on TRAF6-directed development of thymic stroma. Science. (2005) 308:248–51. doi: 10.1126/science.1105677 15705807

[245] Heino M Peterson P Sillanpaa N Guerin S Wu L Anderson G. RNA and protein expression of the murine autoimmune regulator gene (Aire) in normal, RelB-deficient and in NOD mouse. Eur J Immunol. (2000) 30:1884–93. doi: 10.1002/1521-4141(200007)30:7<1884::AID-IMMU1884>3.0.CO;2-P 10940877

[246] Weih F Carrasco D Durham SK Barton DS Rizzo CA Ryseck RP. Multiorgan inflammation and hematopoietic abnormalities in mice with a targeted disruption of RelB, a member of the NF-kappa B/Rel family. Cell. (1995) 80:331–40. doi: 10.1016/0092-8674(95)90416-6 7834753

[247] Burkly L Hession C Ogata L Reilly C Marconi LA Olson D. Expression of relB is required for the development of thymic medulla and dendritic cells. Nature. (1995) 373:531–6. doi: 10.1038/373531a0 7845467

[248] Akiyama T Shimo Y Yanai H Qin J Ohshima D Maruyama Y. The tumor necrosis factor family receptors RANK and CD40 cooperatively establish the thymic medullary microenvironment and self-tolerance. Immunity. (2008) 29:423–37. doi: 10.1016/j.immuni.2008.06.015 18799149

[249] Bonito AJ Aloman C Fiel MI Danzl NM Cha S Weinstein EG. Medullary thymic epithelial cell depletion leads to autoimmune hepatitis. J Clin Invest. (2013) 123:3510–24. doi: 10.1172/JCI65414 PMC3726151 23867620

[250] White AJ Withers DR Parnell SM Scott HS Finke D Lane PJ. Sequential phases in the development of Aire-expressing medullary thymic epithelial cells involve distinct cellular input. Eur J Immunol. (2008) 38:942–7. doi: 10.1002/eji.200738052 18350550

[251] Irla M Hugues S Gill J Nitta T Hikosaka Y Williams IR. Autoantigen-specific interactions with CD4+ thymocytes control mature medullary thymic epithelial cell cellularity. Immunity. (2008) 29:451–63. doi: 10.1016/j.immuni.2008.08.007 18799151

[252] van Ewijk W Shores EW Singer A. Crosstalk in the mouse thymus. Immunol Today. (1994) 15:214–7. doi: 10.1016/0167-5699(94)90246-1 8024681

[253] Philpott KL Viney JL Kay G Rastan S Gardiner EM Chae S. Lymphoid development in mice congenitally lacking T cell receptor alpha beta-expressing cells. Science. (1992) 256:1448–52. doi: 10.1126/science.1604321 1604321

[254] Jenkinson SR Williams JA Jeon H Zhang J Nitta T Ohigashi I. TRAF3 enforces the requirement for T cell cross-talk in thymic medullary epithelial development. Proc Natl Acad Sci U.S.A. (2013) 110:21107–12. doi: 10.1073/pnas.1314859111 PMC3876204 24324158

[255] Reissig S Hovelmeyer N Tang Y Weih D Nikolaev A Riemann M. The deubiquitinating enzyme CYLD regulates the differentiation and maturation of thymic medullary epithelial cells. Immunol Cell Biol. (2015) 93:558–66. doi: 10.1038/icb.2014.122 25601276

[256] Jain R Zhao K Sheridan JM Heinlein M Kupresanin F Abeysekera W. Dual roles for LUBAC signaling in thymic epithelial cell development and survival. Cell Death Differ. (2021) 28:2946–56. doi: 10.1038/s41418-021-00850-8 PMC8481470 34381167

[257] Boehm T. Thymus development and function. Curr Opin Immunol. (2008) 20:178–84. doi: 10.1016/j.coi.2008.03.001 18403191

[258] Manley NR Richie ER Blackburn CC Condie BG Sage J. Structure and function of the thymic microenvironment. Front Biosci (Landmark Ed). (2011) 16:2461–77. doi: 10.2741/3866 21622189

[259] Zuniga-Pflucker JC. T-cell development made simple. Nat Rev Immunol. (2004) 4:67–72. doi: 10.1038/nri1257 14704769

[260] Ma D Wei Y Liu F. Regulatory mechanisms of thymus and T cell development. Dev Comp Immunol. (2013) 39:91–102. doi: 10.1016/j.dci.2011.12.013 22227346

